# Prevalence of urinary schistosomiasis in women: a systematic review and meta-analysis of recently published literature (2016–2020)

**DOI:** 10.1186/s41182-022-00402-x

**Published:** 2022-01-29

**Authors:** Morteza Shams, Sasan Khazaei, Ezatollah Ghasemi, Naser Nazari, Erfan Javanmardi, Hamidreza Majidiani, Saeed Bahadory, Davood Anvari, Mohammad Fatollahzadeh, Taher Nemati, Ali Asghari

**Affiliations:** 1 Zoonotic Diseases Research Center, Ilam University of Medical Sciences, Ilam, Iran; 2 Department of Parasitology, Faculty of Medical Sciences, Tarbiat Modares University, Tehran, Iran; 3 Department of Medical Parasitology, School of Medicine, Dezful University of Medical Sciences, Dezful, Iran; 4 Department of Parasitology and Mycology, School of Medicine, Kermanshah University of Medical Sciences, Kermanshah, Iran; 5 Clinical Research Development Center, “The Persian Gulf Martyrs” Hospital of Bushehr University of Medical Sciences, Bushehr, Iran; 6 Department of Basic Medical Sciences, Neyshabur University of Medical Sciences, Neyshabur, Iran; 7 Department of Parasitology, Student Research Committee, Mazandaran University of Medical Sciences, Sari, Iran; 8 School of Medicine, Iranshahr University of Medical Sciences, Iranshahr, Iran; 9 Department of Parasitology and Mycology, School of Medicine, Isfahan University of Medical Sciences, Isfahan, Iran; 10 Department of Medical Parasitology and Mycology, School of Medicine, Shiraz University of Medical Sciences, Shiraz, Iran

**Keywords:** Epidemiology, Urinary schistosomiasis, Women, Meta-analysis

## Abstract

**Background:**

Urinary schistosomiasis is a serious threat in endemic territories of Africa and the Middle East. The status of female urinary schistosomiasis (FUS) in published literature between 2016 and 2020 was investigated.

**Methods:**

A systematic search in PubMed, Scopus, Google Scholar, and Web of Science, based on the ‘Preferred Reporting Items for Systematic Reviews and Meta-analyses’ checklist, and a meta-analysis using random-effects model to calculate the weighted estimates and 95% confidence intervals (95% CIs) were done.

**Results:**

Totally, 113 datasets reported data on 40,531 women from 21 African countries, showing a pooled prevalence of 17.5% (95% CI: 14.8–20.5%). Most studies (73) were performed in Nigeria, while highest prevalence was detected in Mozambique 58% (95% CI: 56.9–59.1%) (one study). By sample type and symptoms, vaginal lavage [25.0% (95% CI: 11.4–46.1%)] and hematuria 19.4% (95% CI: 12.2–29.4%) showed higher FUS frequency. Studies using direct microscopy diagnosed a 17.1% (95% CI: 14.5–20.1%) prevalence rate, higher than PCR-based studies 15.3% (95% CI: 6.1–33.2%). Except for sample type, all other variables had significant association with the overall prevalence of FUS.

**Conclusions:**

More studies are needed to evaluate the true epidemiology of FUS throughout endemic regions.

**Supplementary Information:**

The online version contains supplementary material available at 10.1186/s41182-022-00402-x.

## Background

Schistosomiasis, due to trematodes of the genus *Schistosoma* (blood flukes), is a snail-transmitted helminthiasis and the third most degenerative tropical disease with substantial morbidity/mortality rates, particularly in low- and middle-income countries [1]. With about 800 million at-risk individuals, schistosomiasis afflicts over 250 million people in tropical and subtropical territories and renders approximately 70 million disability-adjusted life years [1–3]. In endemic areas such as sub-Saharan Africa morbidity is higher among school-aged children (60–80%) than adults (20–40%), with a mortality rate of 280,000 people [4]. Six species out of 24 recognized schistosomes result in disease in humans, comprising *Schistosoma haematobium* (*S. haematobium*) the causative agent of urogenital schistosomiasis (UGS), *S. japonicum*, *S. mansoni*, *S. intercalatum*, *S. mekongi* and *S. guineensis* as agents of hepato-intestinal disease [5]. In a public health perspective, Africa and the Mideast (*S. mansoni* and *S. haematobium*), Southeast Asia (*S. japonicum*) and Latin America (*S. mansoni*) are considered as the most distinguished geographical hotspots for schistosomiasis [6]. Adult paired worms would stay alive in host’s blood stream for about 3–10 years and produce numerous spiny eggs, rendering chronicity and pathologic outcomes of the infection [7–9].

The putative signs and symptoms of UGS were initially ascribed about 1900 Before the Common Era (BCE), when hematuria was a common finding in Egyptian males, referred to as “menstruation” [10]. Infected planorbid snails, *Bulinus* spp., are intermediate hosts releasing motile furcocercous cercariae in surrounding water supplies. Following skin cercarial invasion and migration thorough lungs and liver, *S. haematobium* worms would finally lodge in the genitourinary venous complex, in particular bladder veins, where they mature and copulate therein [11]. Although harsh disease outcomes primarily arise from the T-cell mediated, granulomatous immune responses against tissue-deposited spiny eggs of schistosomes. Such lesions would represent manifestations comprising hematuria, dysuria, itching, pelvic pain, as well as the life-threatening squamous cell carcinoma of the urinary bladder [12, 13]. Additionally, *S. haematobium* is responsible of egg-induced pathological lesions and associated symptoms in both men and women [14, 15].

An active UGS could be detected through observation of eggs in urine sediments and/or tissue biopsies [16]. For the aim of determining hotspots and control strategies, World Health Organization (WHO) has recommended microscopic-based poly-carbonate filter examination for urinary eggs as well as dipstick assays for urinary heme detection [17, 18]. Serodiagnostic assays identifying antibodies against worm antigens may demonstrate valuable credibility in symptomatic travelers, whereas they usually fail to differentiate active or previous infections, unless those employing circulating antigens [19, 20]. An encouraging degree of sensitivity and specificity have been gained in utilization of molecular assays such as polymerase chain reaction (PCR) for schistosome detection in human serum and urine samples [21]. This method is, also, beneficial for vaginal lavage analysis, revealing the likely traits of the genital schistosomiasis [22].

A very large number of female urinary schistosomiasis (FUS) studies were performed during the last two decades [23]. The emphasis of the present systematic review and meta-analysis was on the published literature during the last 5 years (2016–2020), in order to define the latest status of FUS and its prevalence based on examined subgroups. The novel findings of the present study may alert clinicians to the prevalence of this important helminthiasis and its associated consequences on the genitourinary system of infected female individuals.

## Methods

The present systematic review and meta-analysis was accomplished on the basis of Preferred Reporting Items for Systematic Reviews and Meta-analyses (PRISMA) statement [24] (Additional file 1).

### Information sources and systematic searching

Major English databases including Scopus, PubMed, Web of Science and Google Scholar were systematically searched for articles evaluating the prevalence of FUS worldwide and published during a 5-year time period, from January 2016 until the end of 2020. This procedure was conducted using the following keywords alone or in combination, using advanced search option in most databases and Medical Subject Heading (MeSH) option in PubMed databases: “Urinary Schistosomiasis” AND “Prevalence” OR “Epidemiology” AND “Female” OR “Women” Or “Girl”, where “AND” and/or “OR” are Boolean operators. Hand-searching of the bibliographic list of related papers was an additional task to more cover those papers not found via database exploration. Briefly, title and abstract of the literature were accurately reviewed (H.M. and M.F.), relevant papers were included, and upon duplicate removal, full-texts of eligible papers were retrieved (T.N.). Any disagreements were obviated by discussion and consensus with the leading researchers (M.SH and A.A.).

### Inclusion/exclusion criteria and data collection

Specific inclusion criteria were determined in order to thoroughly gather relevant peer-reviewed cross-sectional studies and conference reports limited to women population in a 5-year time period (2016–2020). Only those papers with specified sample size and number of FUS-positive women, diagnosed either by microscopic, filtration, sedimentation and/or molecular techniques were included in current systematic review. Other study types (case reports, letters, reviews), studies evaluating animals or other Schistosomal infections, investigations without sample size/prevalence rates or lacking full-texts were all excluded from the present systematic review and meta-analysis. Finally, a pre-designed Microsoft Excel Spreadsheet^®^ was used to extract the required information (E.J. and S.B.), as follows: first author’s last name, publication year, start and end years of studies, study type, country, province, city, sample type, diagnostic method, sample size, positive number of infected cases and clinical symptoms (hematuria and proteinuria).

### Quality assessment

In the present systematic review, the Newcastle–Ottawa scale was employed to assess the quality of included studies. Those papers with the scores of < 3.5, 3.6–5.25, and 5.26–7 were categorized as low-, moderate-, and high-quality papers, respectively [25].

### Data synthesis and meta-analysis

Meta-analytical approach was done according to previous studies (S.B. and D.A.) using a random-effects model [26–28]. For all included studies, point estimates and their respective 95% confidence intervals (CIs) of weighted prevalence were calculated. Heterogeneity among these studies or variation in study outcomes was visualized by drawing forest plots, calculated by *I*^*2*^ and Cochrane’s *Q* tests [29, 30]. The subgroup analysis was performed based on year, country, sample type, symptoms and diagnostic methods. The presence of publication bias was estimated by using Egger’s regression test [31]. This kind of bias, if present, skews the results and published reports are not a representative sample of the available evidence anymore. The trim-and-fill method was, also, used to “estimate the number of missing studies that might exist in a meta-analysis and the effect that these studies might have had on its outcome” [32]. *P*-values less than 0.05 were considered statistically significant. All analytical functions were applied by Comprehensive Meta-analysis (CMA) version 2.2. (Biostat Inc., USA).

## Results

**Fig. 1:**
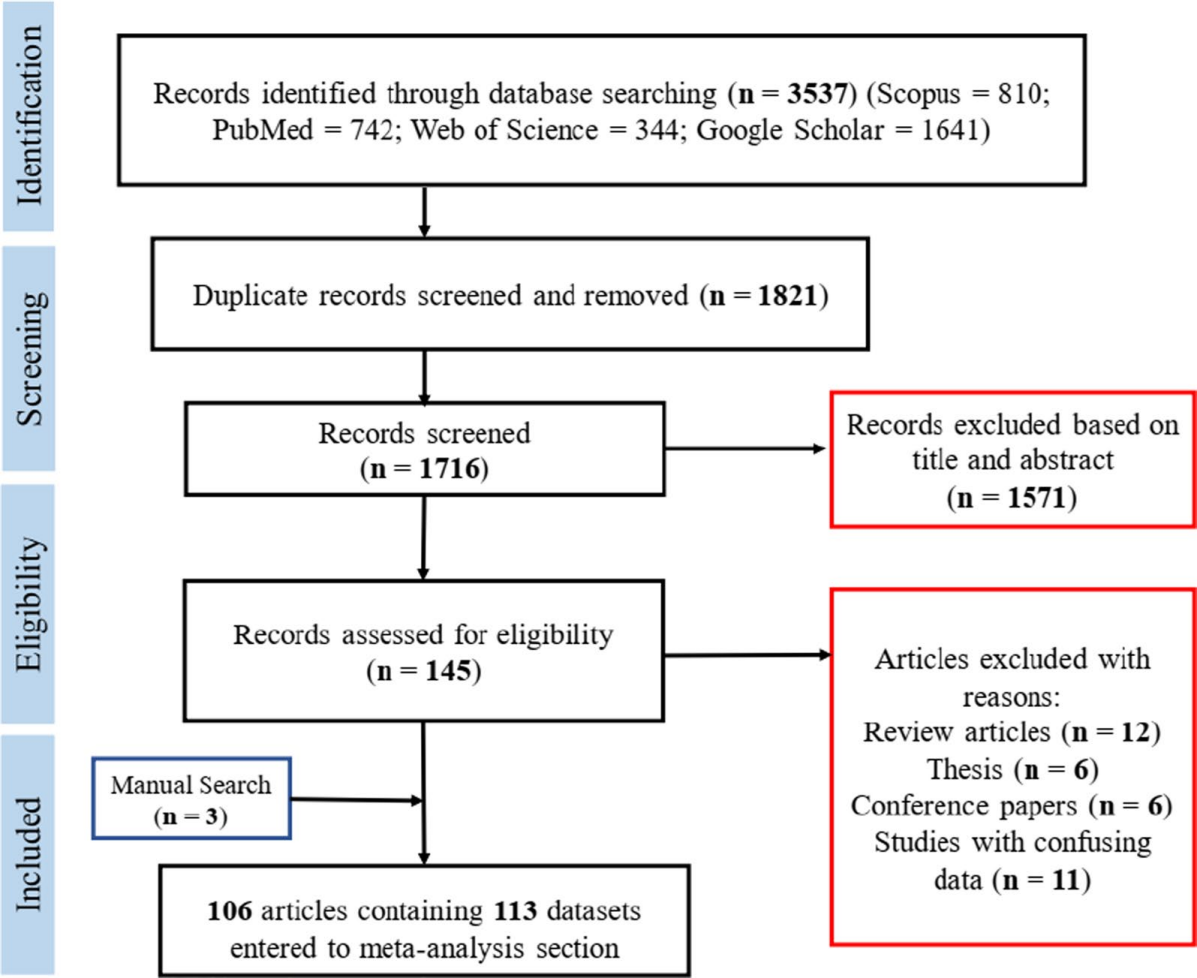
PRISMA flow diagram describing included/excluded studies on FUS prevalence (2016–2020)

The flow diagram of the systematic search process and inclusion of relevant papers is shown in Fig. 1. Initially, 3537 datasets were identified through comprehensive database exploration. After removing duplicates (1821) and those with irrelevant title and abstract (1571), 145 datasets were finally assessed for eligibility. Among these, 35 datasets were excluded with reasons (review papers, theses, conference papers and studies with confusing data) and 3 additional datasets were added through manual searching. Therefore, 106 articles containing 113 datasets were finally included in our meta-analysis (Table 1) [33–139].

**Table 1:** Detailed characteristics of the included studies in the present systematic review and meta-analysis (2016–2020)

No.	References	Country	Province/city	Time of data collection	Sample type	Method	Sample size	Positive no	Quality assessment score
1	Awosolu, 2020 [56]	Nigeria	Osun and Kwara	2012	Urine	Filtrations and microscopic examination	258	122	5
2	Olayinka, 2020 [112]	Nigeria	Ogun	2015–2017	Urine	Microscopic examination	280	42	6
3	Awosolu, 2019 [55]	Nigeria	Ikota	2015	Urine	Microscopic examination	74	20	7
4	Otuneme, 2019 [118]	Nigeria	Ogun	2017	Urine	Microscopic examination	47	39	5
5	Muhammad, 2019 [101]	Nigeria	Sokoto	NR	Urine	Microscopic examination	107	47	5
6	Sule, 2019 [129]	Nigeria	Kano	NR	Urine	Microscopic examination	56	0	6
7	Idris, 2019 [87]	Nigeria	New-Bussa	NR	Urine	Microscopic examination	24	2	7
8	Geraji, 2019 [81]	Nigeria	Jalingo	2019	Urine	Microscopic examination	86	13	7
9	Adamu, 2019 [36]	Nigeria	Kaduna	2017	Urine	Microscopic examination	136	4	7
10	Ngwamah, 2019 [105]	Nigeria	Adamawa	NR	Urine	Microscopic examination	679	141	7
11	Aribodor, 2019 [51]	Nigeria	Enugu	2016	Urine	Microscopic examination	121	17	7
12	Sobande, 2019 [128]	Nigeria	Ogun	NR	Urine	Microscopic examination	84	40	6
13	Obisike, 2019 [110]	Nigeria	Benue	2017	Urine	Membrane filtration and (sedimentation) microscopic examination	84	20	5
14	Ahmed, 2019 [40]	Nigeria	Katsina	NR	Urine	(sedimentation) Microscopic examination	68	15	6
15	Aderibigbe, 2019 [37]	Nigeria	Kwara	NR	Urine	Microscopic examination	883	293	7
16	Noriode, 2018 [106]	Nigeria	Edo	NR	Urine	Microscopic examination	109	75	5
17	Bishop, 2016 [164]	Nigeria	Kaduna	NR	Urine	Microscopic examination	92	5	6
18	Mohammed, 2018 [95]	Nigeria	Sokoto	2016	Urine	Microscopic examination	51	18	5
19	Akinneye, 2018 [43]	Nigeria	Ondo	NR	Urine	Microscopic examination	202	22	5
20	Alabi, 2018 [46]	Nigeria	Ogun	NR	Urine	Microscopic examination	73	36	6
21	Damen, 2018 [68]	Nigeria	Plateau	NR	Urine	Microscopic examination	7	1	6
22	Yauba, 2018 [138]	Nigeria	Maiduguri	2014–2015	Urine	Microscopic examination	180	113	7
23	Abdulkareem, 2018 [34]	Nigeria	Kwara	NR	Urine	Microscopic examination	309	131	7
24	Oladeinde, 2018 [111]	Nigeria	Edo	2014	Urine	Microscopic examination	98	8	6
25	Ebong, 2018 [70]	Nigeria	Akwa Ibom	NR	Urine	Microscopic examination	199	5	7
26	Akeju Adebayo, 2018 [42]	Nigeria	Ondo	NR	Urine	Microscopic examination	1022	441	5
27	Oluwole, 2018 [114]	Nigeria	Ogun	2013	Urine	Microscopic examination	1034	43	6
28	Adewale, 2018 [38]	Nigeria	Ondo	NR	Urine	Microscopic examination	190	44	6
29	Nwachukwu, 2018 [107]	Nigeria	Imo	2014–2016	Urine	Test strip and filtration	1125	57	7
30	Nwachukwu, 2018 [108]	Nigeria	Ebonyi	2016–2017	Urine	Microscopic examination	254	8	7
31	Duwa, 2018 [69]	Nigeria	Kano	2018	Urine	Microscopic examination	105	8	5
32	Babagana, 2018 [57]	Nigeria	Borno	NR	Urine	Microscopic examination	180	31	7
33	Mohammed, 2018 [94]	Nigeria	Kebbi	2016	Urine	(Filtration) Microscopic examination	81	16	5
34	Oluwole, 2018 [114]	Nigeria	Ogun	NR	Urine and vainal lavage	Microscopic and gynecologic examination	317	149	6
35	Kenneth, 2017 [92]	Nigeria	Edo	NR	Urine	Microscopic examination	76	6	7
36	Birma, 2017 [61]	Nigeria	Adamawa	NR	Urine	Microscopic examination	90	42	5
37	Amoo, 2017 [47]	Nigeria	Ogun	NR	Urine	Microscopic examination	160	61	6
38	Paul, 2017 [119]	Nigeria	Cross River	NR	Urine	Microscopic examination	140	24	5
39	Orpin, 2017 [116]	Nigeria	Katsina	NR	Urine	Microscopic examination	145	12	5
40	Ekanem, 2017 [71]	Nigeria	South-South	2011	Urine	Microscopic examination	177	27	6
41	Akpan, 2017 [45]	Nigeria	Cross River	NR	Urine	Microscopic examination	208	34	7
42	Elom, 2017 [73]	Nigeria	Ebonyi	NR	Urine	Microscopic examination	147	33	7
43	Akpan, 2017 [44]	Nigeria	Cross River	NR	Urine	Microscopic examination	122	1	7
44	Abubakar, 2017 [35]	Nigeria	Jigawa	2015	Urine	Microscopic examination	65	46	7
45	Dalhat, 2017 [67]	Nigeria	Sokoto	NR	Urine	Microscopic examination	140	41	7
46	Emmanuel, 2017 [75]	Nigeria	Benue	2014	Urine	Microscopic examination	207	77	6
47	Wokem, 2017 [135]	Nigeria	Abia	NR	Urine	Microscopic examination	570	215	7
48	Anorue, 2017 [49]	Nigeria	Ebonyi	2002–2003	Urine	Microscopic examination	1367	640	6
49	Orpin, 2016 [117]	Nigeria	Benue	NR	Urine	Microscopic examination	104	8	7
50	Onile, 2016 [115]	Nigeria	Eggua	2012–2013	Urine	Microscopic examination	178	45	7
51	Houmsou, 2016 [86]	Nigeria	Taraba	NR	Urine	Microscopic examination	529	231	5
52	Goodhead, 2016 [83]	Nigeria	River	NR	Urine	Microscopic examination	76	17	7
53	Usman, 2016 [133]	Nigeria	Bauchi	NR	Urine	Microscopic examination	300	58	7
54	Dahesh, 2016 [66]	Nigeria	Giza	2016	Urine	Microscopic examination	582	12	7
55	Igbeneghu, 2016 [88]	Nigeria	Osun	2016	Urine	Microscopic examination	154	60	7
56	Nafiu, 2016 [104]	Nigeria	Niger	2016	Urine	Microscopic examination	97	9	6
57	Abah, 2016 [33]	Nigeria	River	2016	Urine	Microscopic examination	184	23	5
58	Umar, 2016 [132]	Nigeria	Kano	NR	Urine	Microscopic examination	20	9	5
59	Atalabi, 2016 [52]	Nigeria	Katsina	NR	Urine	Microscopic examination	240	14	6
60	Houmsou, 2016 [86]	Nigeria	Taraba	NR	Urine	Microscopic examination	510	3	7
61	Nwibari, 2016 [165]	Nigeria	Plateau	NR	Urine	Microscopic examination	134	6	5
62	Omoruyi, 2016 [166]	Nigeria	Edo	NR	Urine	Microscopic examination	77	4	6
63	Morenikeji, 2016 [99]	Nigeria	Ogun	NR	Urine	Microscopic examination	79	60	6
64	Bashir, 2016 [60]	Nigeria	Jigawa	NR	Urine	Microscopic examination	31	2	7
65	Ganau, 2016 [79]	Nigeria	Sokoto	NR	Urine	Microscopic examination	58	24	5
66	Musa, 2016 [102]	Nigeria	Kaduna	NR	Urine	Microscopic examination	131	13	6
67	Ajakaye, 2016 [41]	Nigeria	Ondo	NR	Urine	Microscopic examination	404	50	7
68	Mong, 2016 [98]	Nigeria	Abia	NR	Urine	Microscopic examination	129	13	7
69	Atalabi, 2016 [53]	Nigeria	Katsina	2015	Urine	Microscopic examination	317	23	6
70	Oluwatoyin, 2016 [113]*	Nigeria	Ibadan	NR	Urine	Microscopic examination	507	1	7
71	Oluwatoyin, 2016 [113]	Nigeria	Ibadan	NR	Urine	Microscopic examination	507	28	6
72	Bishop, 2016 [63]	Nigeria	Kaduna	NR	Urine	Microscopic examination	251	39	5
73	Maki, 2020 [93]	Sudan	Darfur	2018	Urine	Microscopic examination	55	39	6
74	Qutoof, 2019 [122]	Sudan	Khartoum	NR	Urine	Microscopic examination	589	2	5
75	Elsiddig, 2019 [74]	Sudan	White Nile	2011	Urine	Microscopic examination	162	67	6
76	Hajissa, 2018 [85]	Sudan	Khartoum	2017–2018	Urine	Microscopic examination	95	11	6
77	Mohammed, 2018 [96]	Sudan	White Nile	NR	Urine	Microscopic examination	175	97	7
78	Talab, 2018 [167]	Sudan	White Nile	2014	Urine	(Filtration) Microscopic examination	174	97	5
79	Sulieman, 2017 [130]	Sudan	River Nile	2016	Urine	(Sedimentation) Microscopic examination	191	1	6
80	Sabah Alzain Mohamed, 2017 [124]	Sudan	El khiar	2016	Urine	Microscopic examination	76	7	5
81	Afifi, 2016 [39]	Sudan	Kassala	2013	Urine	Microscopic examination	1238	172	6
82	Elhusein, 2016 [72]	Sudan	Gezira	2016	Urine	Microscopic examination	29	0	7
83	Shukla, 2019 [126]	South Africa	KwaZulu-Natal	2011–2013	Urine and cervico-vaginal lavage	Microscopic examination	933	256	5
84	Galappaththi-Arachchige, 2018 [78]	South Africa	KwaZulu-Natal	NR	Urine	Microscopic examination	1123	292	5
85	Kabuyaya, 2017 [89]	South Africa	uMkhanyakude	2015	Urine	Microscopic examination	199	73	7
86	Galappaththi-Arachchige, 2016[168]	south Africa	KwaZulu-Natal	NR	Urine	Microscopic examination	883	270	6
87	Pillay, 2016 [169]	South Africa	KwaZulu-Natal	2010–2012	vaginal lavages and Urine	PCR	394	38	7
88		South Africa	KwaZulu-Natal	2010–2012	Urine	PCR	394	91	7
89		South Africa	KwaZulu-Natal	2010–2012	Urine	Microscopic examination	394	78	7
90	Fokuo, 2020 [76]	Ghana	Asutsuare	2014	Urine	Microscopic examination	59	8	6
91	Arhin-Wiredu, 2019 [50]	Ghana	Akyemansa	2014	Urine	Microscopic examination	161	10	6
92	Nyarko, 2018 [109]	Ghana	different municipal-ities	2016	Urine	Microscopic examination	173	7	6
93	Boye, 2016 [65]	Ghana	Apewosika and Putubiw	2013	Urine	Microscopic examination	114	16	5
94	Wilkinson, 2018 [134]	Malawi	Lilongwe	2013	Urine	Microscopic examination	96	2	6
95	Kayuni, 2017 [91]	Malawi	Mangochi	2012	Urine	Microscopic examination	226	29	6
96	Moyo, 2016 [100]	Malawi	Nkhotakota	NR	Urine	Microscopic examination	51	6	6
97	Yameny, 2018 [137]	Egypt	El-Fayoum	NR	Urine	Microscopic examination	487	33	7
98	Ghieth, 2017 [82]	Egypt	Beni Suef	NR	Urine	Microscopic examination	220	0	5
99	Kaiglova, 2020 [90]	Kenya	Kwale	2018	Urine	Microscopic examination	323	47	5
100	Mutsaka-Makuvaza, 2019 [103]	Zimbabwe	Mashonaland	2010	Urine	Microscopic examination	569	96	6
101	Woldegerima, 2019 [136]	Ethiopia	Sanja	2017–2018	Urine	Microscopic examination	189	53	7
102	Phillips, 2018 [120]	Mozambique	Cabo Delgado	2011	Urine	Microscopic examination	7538	4372	7
103	Gbalegba, 2017 [80]	Mauritania	Kaedi	2014–2015	Urine	Microscopic examination	1064	54	6
104	Simoonga, 2017 [127]	Zambia	Lusaka	NR	Urine	Microscopic examination	954	83	7
105	Balahbib, 2017 [58]	Morocco	Tata	2015	Urine	Microscopic examination	13	0	6
106	Anchang-Kimbi, 2017 [48]	Cameroon	Mount Cameroon	2014	Urine	Microscopic examination	250	117	7
107	Mombo-Ngoma, 2017 [97]	Gabon	Lambarene	2009–2013	Urine	Microscopic examination	1115	103	7
108	Greter, 2016 [84]	Chad	Chad	NR	Urine	(Filtration) Microscopic examination	96	1	7
109	Botelho, 2016 [64]	Guinea-Bissau	Guinea-Bissau	NR	Urine	Microscopic examination	43	8	6
111	Senghor, 2016 [125]	Senegal	Niakhar	2011–2014	Urine	Microscopic examination	320	149	5
111	Rasomanamihaja, 2016 [123]	Madagascar	Madagascar	2015	Urine	Microscopic examination	1043	325	5
112	Bangura, 2016 [59]	Sierra Leon	Korwama and Lewabu	2015	Urine	Microscopic examination	86	32	7
113	Zida, 2016 [139]	Burkina Faso	Bazega	2013	Urine	Microscopic examination	151	7	7

*In this dataset, *S. mansoni* was found in urine instead of *S. haematobium*

**Fig. 2:**
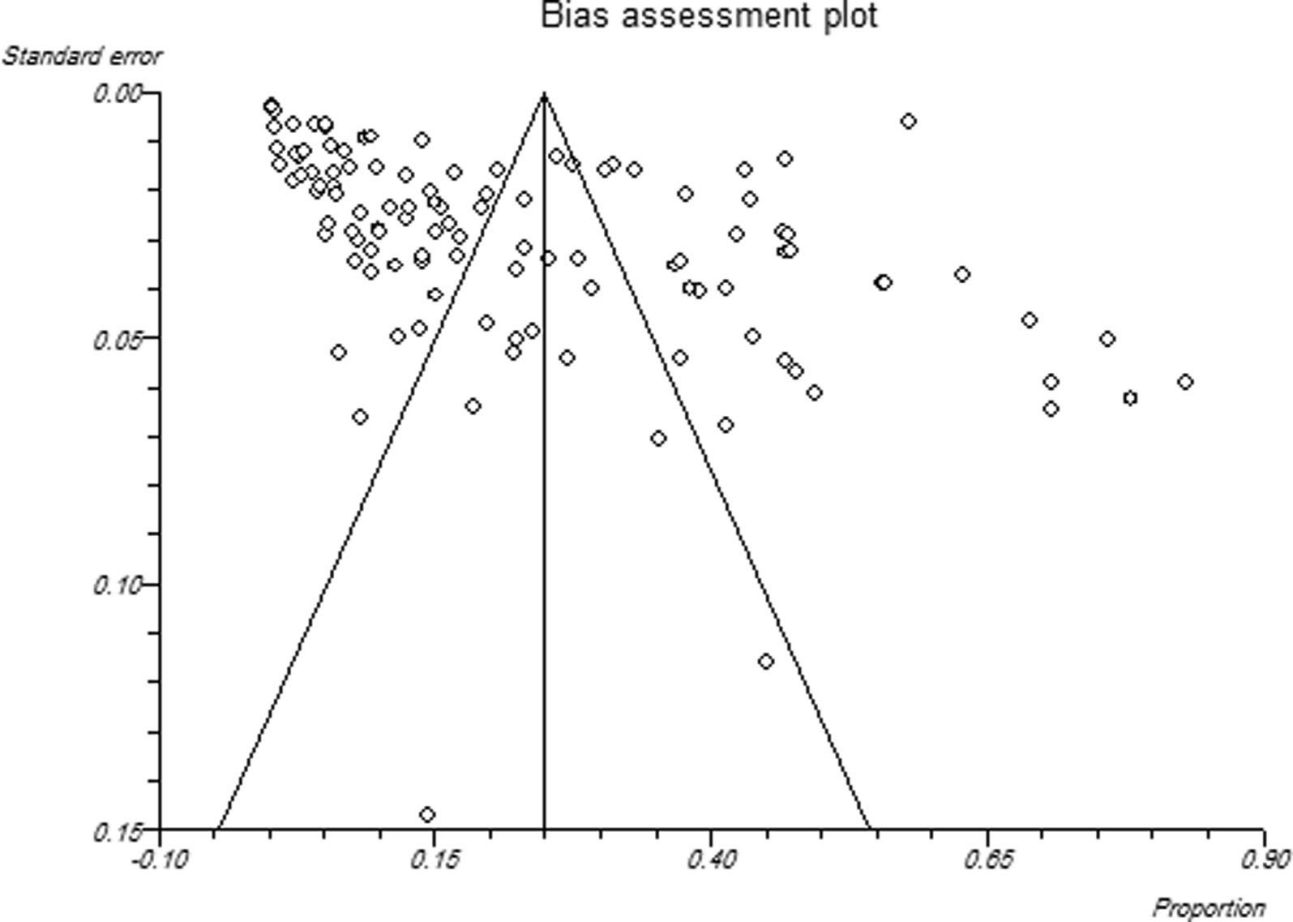
A bias assessment plot from Egger for the FUS prevalence (2016–2020)

**Fig. 3:**
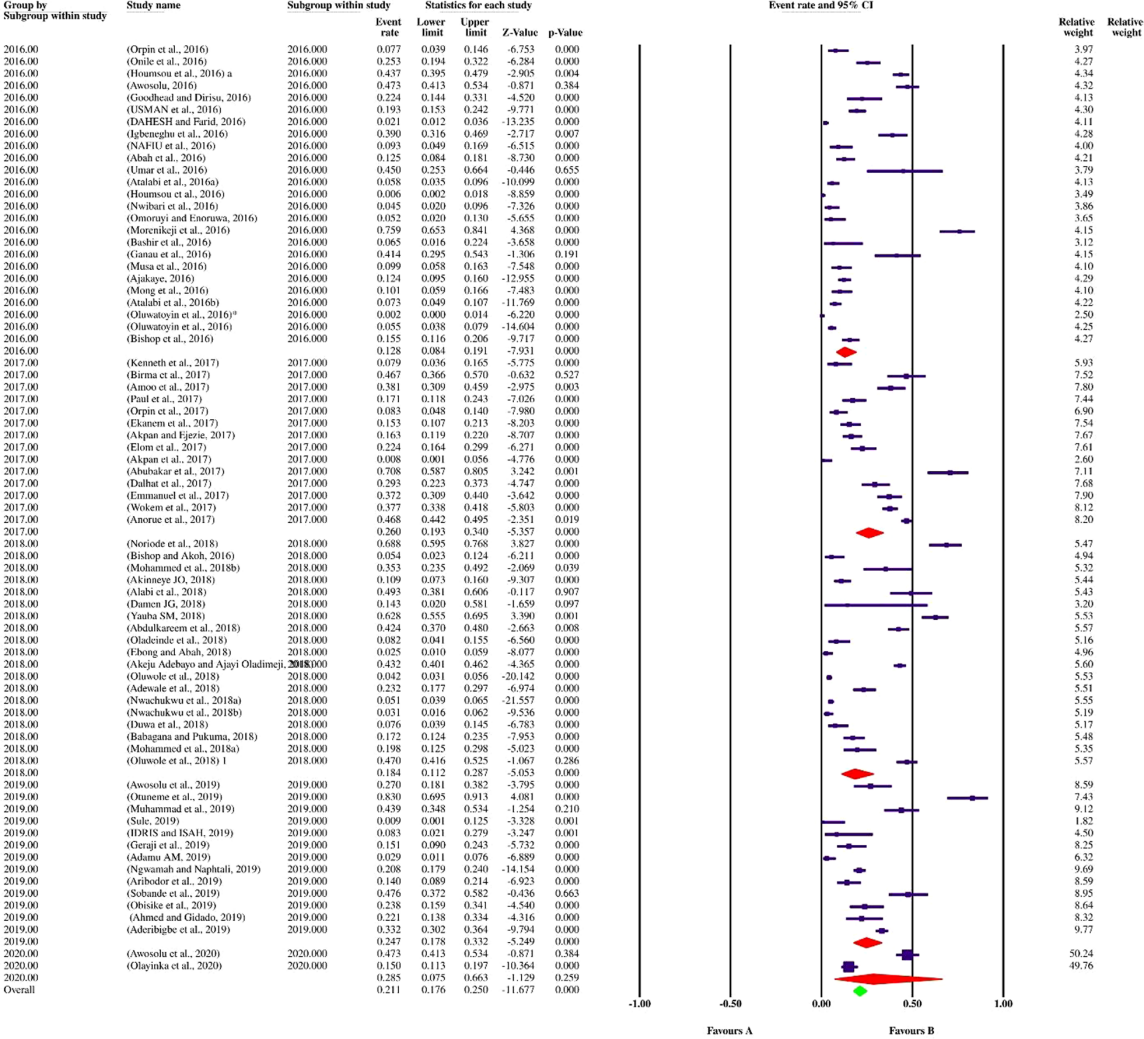
Forest plot of year-based prevalence in Nigeria (2016–2020)

**Fig. 4:**
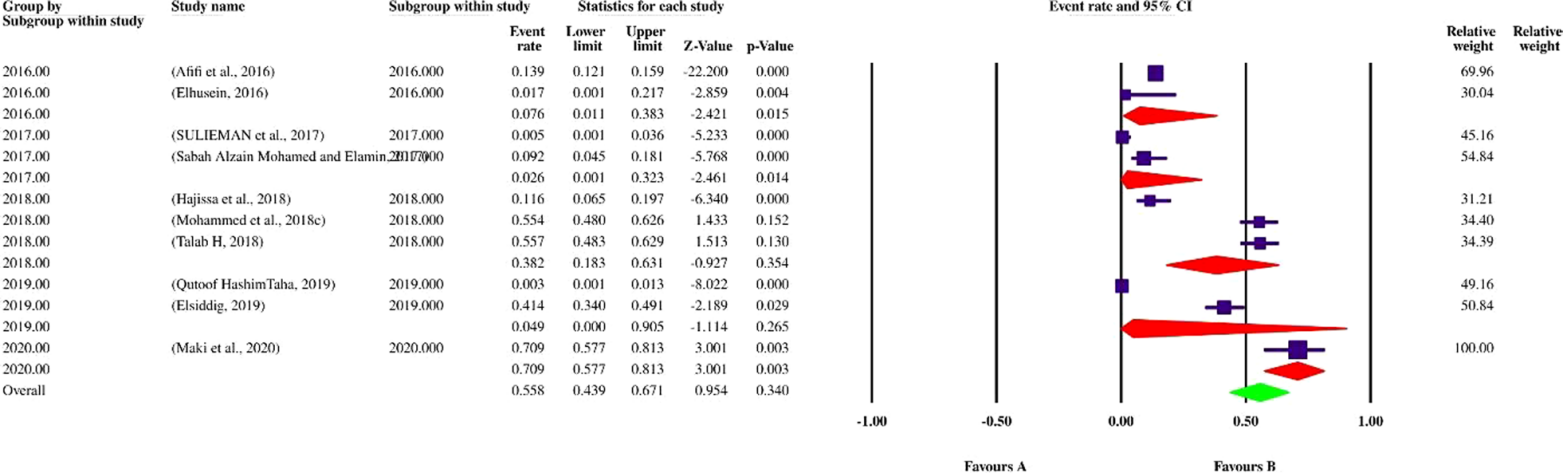
Forest plot of year-based prevalence in Sudan (2016–2020)

**Fig. 5:**
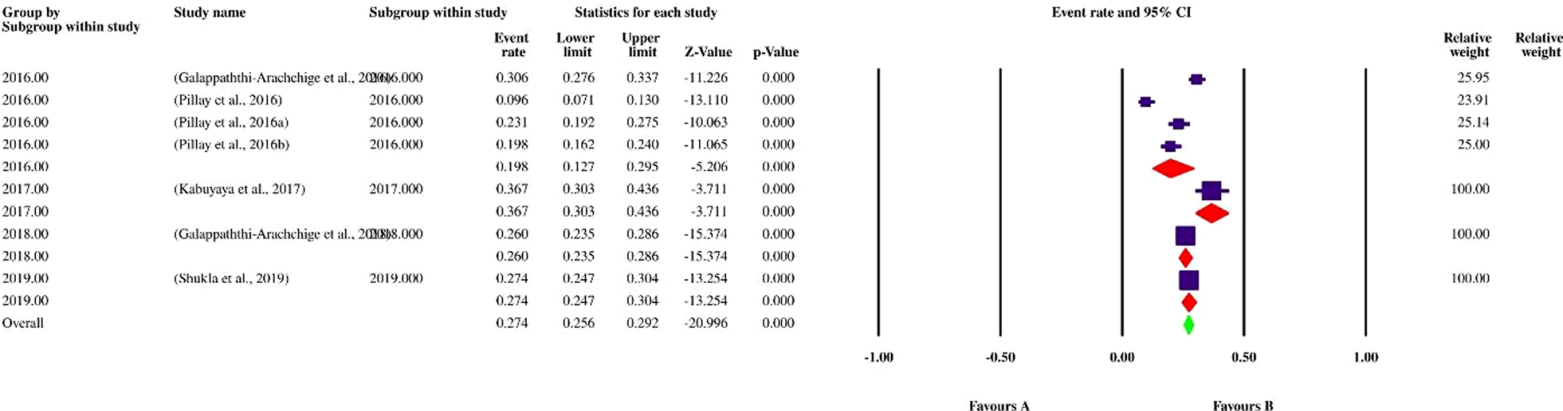
Forest plot of year-based prevalence in South Africa (2016–2020)

**Fig. 6:**
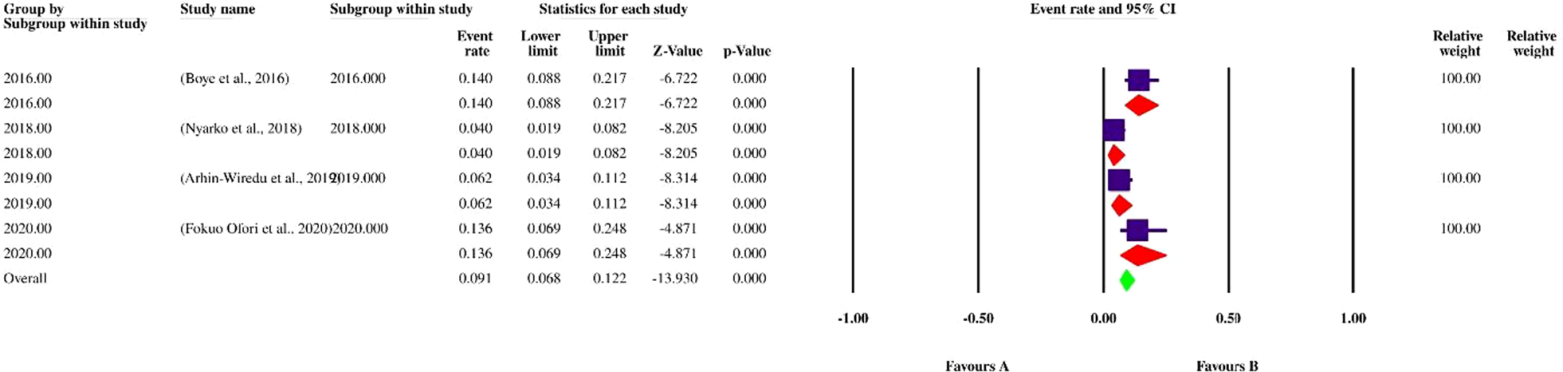
Forest plot of year-based prevalence in Ghana (2016–2020)

**Fig. 7:**
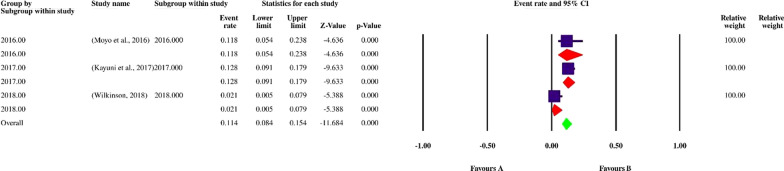
Forest plot of year-based prevalence in Malawi (2016–2020)

**Fig. 8:**
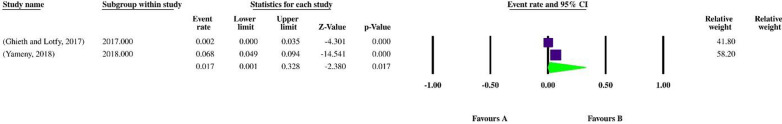
Forest plot of year-based prevalence in Egypt (2016–2020)

Finally, 113 datasets evaluating 40,531 individuals were included in the present review. Among these, 11,308 individuals were shown to be affected by FUS and based on the random-effects model meta-analysis, the pooled prevalence of FUS was 17.5% (95% CI: 14.8–20.5%). The included studies demonstrated a strong heterogeneity (*I*^2^ = 98.12%, *P* < 0.01) (Additional file 2). Publication bias was checked by Egger’s regression test, showed that it may have a substantial impact on total prevalence estimate (Egger’s bias: 7.5, *P* < 0.01) (Fig. 2). Since the heterogeneity of included studies was very high, meta-regression of subgroups such as year, country, type of sample, type of symptoms, and diagnostic method were used to overcome heterogeneity (Table 2). According to subgroup analysis of included data, the prevalence of FUS demonstrated a relatively but worrying increasing trend from 14.6% (95% CI: 11.3–18.6%) in 2016 to 28.6% (95% CI: 13.1–51.6%) in 2020, respectively. In total, studies were conducted in 21 countries, including Nigeria (73 datasets), Sudan (10 datasets), South Africa (7 datasets), Ghana (4 datasets), Malawi (3 datasets), Egypt (2 datasets), as well as Kenya, Zimbabwe, Ethiopia, Mozambique, Mauritania, Zambia, Morocco, Cameroon, Gabon, Chad, Guinea-Bissau, Senegal, Madagascar, Sierra Leone and Burkina Faso (one dataset per country). The highest prevalence rates were estimated for women in Mozambique with 58% (95% CI: 56.9–59.1%) (one study), while female individuals in Chad had the lowest prevalence rate 1.0% (95% CI: 0.1–7.0%). Year-based prevalence for the six most studied countries, showed no determined pattern for frequency of FUS, however, a relatively decreasing pattern of prevalence was recorded for Malawi (three studies) (Figs. 3, 4, 5, 6, 7, 8). Regarding sample type, urine and vaginal lavage were gathered from examined women, with vaginal lavage demonstrating a higher frequency of FUS [25.0% (95% CI: 11.4–46.1%)] than urine specimen [17.2% (95% CI: 14.5–20.3%)]. Reportedly, hematuria and proteinuria as the most prominent symptoms of FUS were estimated in some studies, showing 19.4% (95% CI: 12.2–29.4%) and 13.6% (95% CI: 6.69–24.8%) prevalence rates, correspondingly. Direct microscopy was the most frequently utilized diagnostic test, yielding a relatively higher prevalence 17.1% (95% CI: 14.5–20.1%) than PCR method 15.3% (95% CI: 6.1–33.2%); however, only two studies employed molecular method. Additional microscopy-based procedures were filtration and sedimentation, which in detail yielded a prevalence rate of 18.2% (95% CI: 5.9–43.9%) and 11.4% (95% CI: 3.6–30.9%), respectively. Altogether, subgroup analysis revealed that there were statistically significant differences between the overall prevalence of FUS and year. Of note, the quality score of the included papers is provided in Additional file 3.

**Table 2:** Subgroup analysis of FUS prevalence according to year, country, type of sample, type of symptoms and diagnostic methods

Subgroup variable	Prevalence % (95% CI)	*I*^2^ (%)	Heterogeneity (*Q*)	*P*-value	Interaction test (*X*^*2*^)	*P*-value
Year						
2016	14.6 (11.3–18.6)	96.3%	1034.7	< 0.01	375.3	< 0.01
2017	17.5 (12–24.9)	97.8%	1055.2	< 0.01		
2018	19.0 (13.1–26.7)	98.8%	2179.6	< 0.01		
2019	21.7 (16.8–27.5)	93.4%	274.7	< 0.01		
2020	28.6 (13.1–51.6)	97.1%	138.2	< 0.01		
Country						
Ghana	9.1 (6.8–12.2)	73.46%	11.31	< 0.01		
Malawi	11.4 (0.8–15.4)	70.62%	6.81	< 0.01		
Nigeria	21.1 (17.6–25.0)	96.9%	2337.91	< 0.01		
South Africa	27.4 (25.6–29.2)	92.53%	80.36	< 0.01		
Sudan	55.8 (43.9–67.1)	97.59%	374.17	< 0.01	430.6	< 0.01
Egypt	1.7 (0.1–32.8)	83.57	5.90	< 0.01		
Type of sample						
Urine	17.2 (14.5–20.3)	98.11%	5949.4	< 0.01	1285.2	> 0.05
Vaginal lavage	25.0 (11.4–46.1)	98.2%	110.40	< 0.01		
Type of symptoms						
Hematuria	19.4 (12.2–29.4)	92.33%	52.19	< 0.01	82.4	< 0.01
Proteinuria	13.6 (6.69–24.8)	–	0.00	= 1.00		
Diagnostic method						
Direct microscopy	17.1 (14.5–20.1)	98.1%	6013	< 0.01	350.6	< 0.01
Filtration and microscopy	18.2 (5.9–43.9)	99.1%	563.1	< 0.01		
PCR	15.3 (6.1–33.2)	95.9%	24.64	< 0.01		
Sedimentation and microscopy	11.4 (3.6–30.9)	96.6%	59.5	< 0.01

## Discussion

Helminth-induced diseases are ancient catastrophic phenomena in humans, some dating back to pre-biblical era, with huge but chronic and snaky damages in nature [140]. Schistosomiasis or bilharziasis is one of the most important water-borne helminthic diseases, which have always been interconnected with archaic civilizations over the millennia, and it is still a global public health concern due to its astonishing, complex life cycle [141, 142]. Among schistosome species infecting humans, *S. haematobium* worms are the causative agents of UGS which localize within draining venous complex of the pelvic organs such as uterus, cervix and the bladder [143]. These worms are highly prolific, releasing about 3000 eggs/day, half of which are excreted through urine, while the rest are lodged within vasculature of urogenital organs. Immune-mediated pathologic processes elicited against tissue-embedded ova result in granulomatous inflammation, tissue destruction and the so-called “sandy patches” as fibrotic nodules [16]. With respect to the significance of UGS and large number of affected individuals, the present systematic review and meta-analysis was contrived in order to reveal the latest status of urinary schistosomiasis in women population based on published literature in the last 5 years and provide a premise for future clinical directions on women health.

The required information was assembled from available full-texts published between 2016 and 2020 and their overall estimates were assessed through a meticulous meta-analytical method. During last 5 years, 11,308 out of 40,531 women were suffering from urinary schistosomiasis, contributing to the global weighted prevalence of 17.5% (95% CI: 14.8–20.5%). Interestingly, all cases in the last 5 years were from African countries. This continent is probably known as the “cradle of schistosomes”, since African great lakes provide a favorable milieu for the optimum evolution of both parasites and their respective intermediate hosts [144]. Schistosomiasis may have spread to Africa, particularly Egypt, in virtue of monkey importation and slave trades during fifth dynasty of pharaohs [145]. Based on our results obtained from limited number of heterogeneous investigations included in the present meta-analysis, a large number of studies (73) on FUS were done in a western African nation, Nigeria, whereas the highest prevalence rate was estimated for women in Mozambique with 58% (95% CI: 56.9–59.1%) (one study), a country in the southeast coast of Africa. Nigerian researchers have shown a substantial effort in search of urinary schistosomiasis during last 5 years by conducting 73 datasets, which could be a favorable layout for other African countries [143]. Nevertheless, the true picture of FUS prevalence throughout African territories in a 5-year time period was not accurately captured, since out of 21 countries examining female individuals, only 6 countries had sufficient studies to perform meta-analytical approach and most of the remaining had only one investigation per country. Moreover, a statistically significant gradual increase was observed in FUS prevalence based on publication year of the included literature, from 2016 until the end of 2020, ranging from 14.6% (95% CI: 11.3–18.6%) to 28.6% (95% CI: 13.1–51.6%), respectively. However, no such an increasing trend was observed in year-based analysis of each country; even the prevalence relatively decreased in Malawi, though only three studies were involved in this country. Such findings derived from limited number of included studies in current review may be interpreted as a spread of the endemic situation of FUS, or as a result of the increased understanding about FUS among health care professionals in each country. Nevertheless, more in-depth studies are required to further elucidate this issue.

The characteristic symptoms of UGS were prominently reported among examined women, so that a higher prevalence rate was recorded for hematuria with 19.4% (95% CI: 12.2–29.4%), in comparison to 13.6% (95% CI: 6.69–24.8%) frequency of proteinuria. As previously mentioned, disease morbidity largely results from entrapped eggs, which strongly induce a granulomatous immune response [146], characterized by Th2-type lymphocytes, alternatively activated macrophages and eosinophils [147, 148]. Thereby, the eggs are immunologically confined within the so-called “granulomas”, containing proteolytic enzymes of egg origin that barricade tissue necrosis [149]. In accordance with our finding, hematuria is considered as a defining symptom in *S. haematobium* infection, mostly being accompanied by suprapubic ailment, burning micturition as well as frequent urination [150]. Poor immunoregulatory mechanisms in response to eggs provoke a lasting fibrotic reaction in the urinary tract of infected individuals [151]. The resulting obstructive uropathy elicit subsequent dreadful consequences such as the hydroureter and hydronephrosis [152]. The latter is the milestone in ascending bacterial superinfections, renal dysfunctions and the ensuing proteinuria [153]. The consequences are more horrific in affected women, since the proximity of vesical and genital venous plexuses facilitates easy migration of parasites and/or eggs, leading to harsh outcomes regarding women’s reproductive health [154–156]. The subsequent lesions in genital organs, from ovaries to vagina, may be associated with pain and stress, allowing human immunodeficiency virus-1 (HIV-1) to simply access sub-epithelial target cells [157]. In a recently published meta-analysis, the chance of acquiring HIV among people suffering from schistosomiasis was 2.3-fold (95% CI: 1.2–4.3%) higher than non-infected patients [158]. Finally, the affected women might experience painful intercourse (dyspareunia), fibrotic ovaries and/or granuloma-induced tubal blockage, all of which lead to the female infertility. Hence, FUS may lead to harsh reproductive outcomes that ultimately endangers the fecundity, fertility and pregnancy of women [159].

The result of the present meta-analysis highlighted that a higher prevalence of FUS was demonstrated by vaginal lavage [25.0% (95% CI: 11.4–46.1%)] than urine specimens [17.2% (95% CI: 14.5–20.3%)]. Although there was not statistically significant difference between the total prevalence of FUS and sample type (*P* > 0.05). Moreover, the results of current review demonstrated that microscopy 17.1% (95% CI: 14.5–20.1%) contributed more to reveal the FUS prevalence than PCR method 15.3% (95% CI: 6.1–33.2%); nevertheless, only two studies utilized molecular method for diagnosis, and any deductions should accompany with caution. Notably, urine filtration (about 10 mL) that is routinely performed for egg detection was more efficient in detecting parasite eggs than sedimentation method, with 18.2% (95% CI: 5.9–43.9%) versus 11.4% (95% CI: 3.6–30.9%), respectively. Urine microscopy is the gold standard in detection of *S. haematobium* eggs in areas of endemicity [160]. However, it is not sensitive sufficiently for monitoring praziquantel therapeutic efficiency in mass drug administration (MDA) campaigns, particularly in low-transmission intensity areas, because weeks after adult worm elimination eggs are still observable in urine or some worms may have temporarily stopped shedding eggs [161]. Also, it lacks adequate sensitivity, due to the fact that eggs are only detectable in urine samples 2 months after infection onwards [162]. Therefore, it is highly recommended to carry out at least two follow-up visits and microscopic examination for more accurate diagnosis [163]. Additionally, in order to enhance the sensitivity and specificity and deter underestimation of the true disease burden, performing highly sensitive methods such as molecular techniques are inevitable [21]. As mentioned earlier, only two studies in the last 5 years used PCR method, which exhibited a remarkable prevalence rate for FUS, implicating the importance of such modalities in accurate detection of urinary schistosomiasis.

The present systematic review and meta-analysis met some limitations, including: (1) lack of adequate prevalence studies in countries other than Nigeria; (2) diagnosis of the infection mostly based on microscopic examination of urine samples; (3) inadequate number of molecular-based studies in the last 5 years, and (4) due to the nature of the systematic review and meta-analysis studies, which exclude some papers relied on a designed inclusion criteria, the provided results are only based on the information extracted from 113 datasets and any definite inference must accompany with caution. Inevitably, implementation of large-scale or nation-wide prevalence studies on FUS throughout African nations, particularly in neglected regions of the continent, using microscopy of urine specimen (gold standard method) coupled with unprecedented molecular approaches will more elucidate the true epidemiological picture of urinary schistosomiasis among women population. Consequently, such information benefits the clinicians for the prevention of the horrible sequelae of chronic FUS.

## Conclusion

In conclusion, information provided in the present systematic review and meta-analysis showed that women in endemic territories in Africa are moderately at risk of acquiring FUS and its harsh consequences, including renal dysfunction, urinary bladder carcinoma as well as reproductive disorders such as dyspareunia and granuloma-induced infertility. Consequently, health assessment of FUS should be considered as a routine necessity for women in susceptible age groups such as those in active reproductive status and/or child-bearing age. Relying only on low-sensitivity microscopic results cannot rule out the presence of schistosomes in blood vessels. Hence, clinical assessment must be performed using gold standard methods, i.e., microscopic examination of urine samples, combined with highly sensitive and specific molecular approaches. Altogether, our goal on better control and prevention of urinary schistosomiasis may not be achievable, unless by a global collaboration to accurately reveal the parasite epidemiology in endemic territories.

## Supplementary Information

**Additional file 1.** PRISMA checklist employed for the present systematic review.

**Additional file 2.** Forest plot of the FUS prevalence obtained from published literature during 2016–2020.

**Additional file 3. **Quality assessment analysis of the included papers using Newcastle–Ottawa scale.

## Data Availability

The dataset(s) supporting the conclusions of this article is(are) included within the article (and its additional files).

## Abbreviations

UGS: Urogenital schistosomiasis
BCE: Before common era
WHO: World Health Organization
PCR: Polymerase chain reaction
FUS: Female urinary schistosomiasis
PRISMA: Preferred Reporting Items for Systematic Reviews and Meta-analyses
MeSH: Medical subject heading
CI: Confidence interval
CMA: Comprehensive meta-analysis
HIV-1: Human immunodeficiency virus-1
MDA: Mass drug administration

## References

[1] Colley DG, Bustinduy AL, Secor WE, King CH (2014). Human schistosomiasis. The Lancet.

[2] Ross AG, Chau TN, Inobaya MT, Olveda RM, Li Y, Harn DA. A new global strategy for the elimination of schistosomiasis. Elsevier; 2017. 10.1016/j.ijid.2016.09.023 27939558

[3] Frahm S, Anisuzzaman A, Prodjinotho UF, Vejzagić N, Verschoor A, Prazeres da Costa C (2019). A novel cell-free method to culture *Schistosoma mansoni* from cercariae to juvenile worm stages for in vitro drug testing. PLoS Negl Trop Dis.

[4] Anisuzzaman, Tsuji N. Schistosomiasis and hookworm infection in humans: disease burden, pathobiology and anthelmintic vaccines. Parasitol Int. 2020:102051. 10.1016/j.parint.2020.102051 31911156

[5] Rollinson D (2009). A wake up call for urinary schistosomiasis: reconciling research effort with public health importance. Parasitology.

[6] Siddiqui AA, Siddiqui SZ (2017). Sm-p80-based schistosomiasis vaccine: preparation for human clinical trials. Trends Parasitol.

[7] Chabasse D, Bertrand G, Leroux J, Gauthey N, Hocquet P (1985). Developmental bilharziasis caused by *Schistosoma mansoni* discovered 37 years after infestation. Bulletin de la Societe de pathologie exotique et de ses filiales.

[8] Warren KS, Mahmoud AA, Cummings P, Murphy DJ, Houser HB (1974). Schistosomiasis mansoni in Yemeni in California: duration of infection, presence of disease, therapeutic management. Am J Trop Med Hyg.

[9] Anisuzzaman SF, Prodjinotho UF, Bhattacharjee S, Verschoor A, da Costa CP. Host-specific serum factors control the development and survival of *Schistosoma mansoni*. Front Immunol. 2021;12. 10.3389/fimmu.2021.635622 PMC8103320 33968028

[10] Ansari N (1973). Epidemiology and control of schistosomiasis (bilharziasis).

[11] Grevelding CG, Langner S, Dissous C (2018). Kinases: molecular stage directors for schistosome development and differentiation. Trends Parasitol.

[12] McManus DP, Dunne DW, Sacko M, Utzinger J, Vennervald BJ, Zhou X-N (2018). Schistosomiasis. Nat Rev.

[13] Ritter M, Gross O, Kays S, Ruland J, Nimmerjahn F, Saijo S (2010). *Schistosoma mansoni* triggers Dectin-2, which activates the Nlrp3 inflammasome and alters adaptive immune responses. Proc Natl Acad Sci.

[14] Kameh D, Smith A, Brock MS, Ndubisi B, Masood S (2004). Female genital schistosomiasis: case report and review of the literature. South Med J.

[15] Kayuni S, Lampiao F, Makaula P, Juziwelo L, Lacourse EJ, Reinhard-Rupp J (2019). A systematic review with epidemiological update of male genital schistosomiasis (MGS): a call for integrated case management across the health system in sub-Saharan Africa. Parasite Epidemiol Control..

[16] Santos LL, Santos J, Gouveia MJ, Bernardo C, Lopes C, Rinaldi G (2021). Urogenital schistosomiasis—history, pathogenesis, and bladder cancer. J Clin Med.

[17] Organization WH. The control of schistosomiasis: second report of the WHO Expert Committee [meeting held in Geneva from 8–15 November 1991]: World Health Organization; 1993. 8322462

[18] Organization WH. Preventive chemotherapy in human helminthiasis. Coordinated use of anthelminthic drugs in control interventions: a manual for health professionals and programme managers: World Health Organization; 2006.

[19] Colley DG, Binder S, Campbell C, King CH, Tchuenté L-AT, Noran EK (2013). A five-country evaluation of a point-of-care circulating cathodic antigen urine assay for the prevalence of *Schistosoma mansoni*. Am J Trop Med Hygiene..

[20] Mott K, Dixon H (1982). Collaborative study on antigens for immunodiagnosis of schistosomiasis. Bull World Health Organ.

[21] Ajibola O, Gulumbe BH, Eze AA, Obishakin E (2018). Tools for detection of schistosomiasis in resource limited settings. Med Sci.

[22] Kjetland EF, Ten Hove RJ, Gomo E, Midzi N, Gwanzura L, Mason P (2009). Schistosomiasis PCR in vaginal lavage as an indicator of genital *Schistosoma haematobium* infection in rural Zimbabwean women. Am J Trop Med Hyg.

[23] Barsoum RS (2013). Urinary schistosomiasis. J Adv Res.

[24] Moher D, Liberati A, Tetzlaff J, Altman DG, Group P (2009). Preferred reporting items for systematic reviews and meta-analyses: the PRISMA statement. PLoS Med.

[25] Anvari D, Pourmalek N, Rezaei S, Fotovati A, Hosseini SA, Daryani A, et al. The global status and genetic characterization of hydatidosis in camels (Camelus dromedarius): a systematic literature review with meta-analysis based on published papers. Parasitology. 2021:1–54. 10.1017/S0031182020001705 PMC11010127 32940199

[26] Ghasemi E, Shamsinia S, Taghipour A, Anvari D, Bahadory S, Shariatzadeh SA (2020). Filarial worms: a systematic review and meta-analysis of diversity in animals from Iran with emphasis on human cases. Parasitology.

[27] Javanmardi E, Majidiani H, Shariatzadeh SA, Anvari D, Shamsinia S, Ghasemi E, et al. Global seroprevalence of Neospora spp. in horses and donkeys: a systematic review and meta-analysis. Veterinary Parasitol. 2020:109299. 10.1016/j.vetpar.2020.109299 33227673

[28] Khademvatan S, Majidiani H, Khalkhali H, Taghipour A, Asadi N, Yousefi E (2019). Prevalence of fasciolosis in livestock and humans: a systematic review and meta-analysis in Iran. Comp Immunol Microbiol Infect Dis.

[29] Cochran WG (1954). The combination of estimates from different experiments. Biometrics.

[30] Higgins JP, Thompson SG, Deeks JJ, Altman DG (2003). Measuring inconsistency in meta-analyses. BMJ (Clinical Research ed).

[31] Egger M, Smith GD (1995). Misleading meta-analysis. BMJ (Clinical Research ed).

[32] Duval S, Tweedie R (2000). Trim and fill: a simple funnel-plot-based method of testing and adjusting for publication bias in meta-analysis. Biometrics.

[33] Abah AE, Onoja H, Nduka FO, Arene FO (2016). Current status of urinary schistosomiasis and some pre-disposing factors in Emelego Community, Rivers State, Nigeria. Acta Parasitol Globalis.

[34] Abdulkareem BO, Habeeb KO, Kazeem A, Adam AO, Samuel UU (2018). urogenital schistosomiasis among school children and the associated risk factors in selected rural communities of Kwara State, Nigeria. J Trop Med.

[35] Abubakar S, Zakariya M, Ahmad MK, Abdullahi MK, Yunusa I (2017). Co-hort study of urinary schistosomiasis among two villages residing along Hadejia Valley, Jigawa State, Nigeria. Bayero J Pure Appl Sci.

[36] DA Adamu AM, Akefe OI, Alimi YA, Adikwu AA, Idoko SI, Kore M, Okita AO, Yikawe SS, Bello SG, Lamai RS, Kolo RL (2019). Epidemiology of urinary schistosomiasis among secondary school students in Kaduna State, Nigeria. J Commun Med Health Educ.

[37] Aderibigbe SA, Okpareke O, Adaramola SO (2019). Diagnosis of urinary schistosomiasis among primary school pupils in Patigi local government: haematuria vs microscopy. Res J Health Sci.

[38] Adewale B, Mafe MA, Sulyman MA, Idowu ET, Ajayi MB, Akande DO (2018). Impact of single dose praziquantel treatment on *Schistosoma haematobium* infection among school children in an Endemic Nigerian Community. Korean J Parasitol.

[39] Afifi A, Ahmed AA, Sulieman Y, Pengsakul T (2016). Epidemiology of schistosomiasis among villagers of the New Halfa Agricultural Scheme, Sudan. Iran J Parasitol.

[40] Ahmed A, Gidado SM (2019). Prevalence of schistosomiasis among schoolchildren in Iyatawa and Faduma communities, Rimi local government area, Katsina state. Katsina J Natl Appl Sci.

[41] Ajakaye OO (2016). Endemicity of urinary schistosmiasis in Ile Oluji/Oke Igbo Local Government Area of Ondo State. Dev Country Stud.

[42] Akeju Adebayo V, Ajayi OJ (2018). Socioeconomic and prevalence of urinary schistosomiasis infection in Riverine Areas of Ondo State, Nigeria. Int J Trop Dis Health.

[43] Akinneye JOFM, Afolabi OJ, Adesina FP (2018). Prevalence of urinary schistosomiasis among secondary school students in Ifedore Local Government, Ondo State Nigeria. Int J Trop Dis.

[44] Akpan SS, Dike PC, Mbah M (2017). The prevalence of urinary schistosomiasis among school children in Nkarasi and Edor communities in Ikom Local Government Area of Cross River State, Nigeria. Pyrex J Med Med Sci.

[45] Akpan SS, Ejezie GC (2017). The prevalence of urinary schistosomiasis in Awi, Akamkpa local government area of cross river state, Nigeria. Int J Curr Sci Technol.

[46] Alabi P, Oladejo SO, Odaibo AB (2018). Prevalence and intensity of urinary schistosomiasis in Ogun state, Southwest, Nigeria. J Public Health Epidemiol.

[47] Amoo KJ, Amoo OA, Oke AA, Adegboyega TT (2017). Prevalence of Urinary Tract Infection (UTI) and concomitant urinary Schistosomiasis among Primary School Children in Remo North Local Government, Ogun State, Nigeria. IOSR J Dent Med Sci.

[48] Anchang-Kimbi JK, Elad DM, Sotoing GT, Achidi EA (2017). Coinfection with *Schistosoma haematobium* and *Plasmodium falciparum* and Anaemia severity among pregnant women in Munyenge, Mount Cameroon Area: a cross-sectional study. J Parasitol Res.

[49] Anorue CO, Nwoke BEB, Ukaga CN (2017). The incidence of urinary Schistosomiasis in Ohaukwu local Government Area of Ebonyi. Asian J Biomed Pharm Sci.

[50] Arhin-Wiredu K, Kumi AA, Quarshie SS, Tawiah PA, Oduro EA, Hotorvi C, et al. Prevalence and associated factors of urinary Schistosomiasis among basic school children in the Akyemansa District, Ghana. Asian J Med Health. 2019:1–10.

[51] Aribodor DN, Bassey SA, Yoonuan T, Sam-Wobo SO, Aribodor OB, Ugwuanyi IK (2019). Analysis of Schistosomiasis and soil-transmitted helminths mixed infections among pupils in Enugu State, Nigeria: implications for control. Infect Dis Health.

[52] Atalabi TE, Lawal U, Akinluyi FO (2016). Urogenital schistosomiasis and associated determinant factors among senior high school students in the Dutsin-Ma and Safana Local Government Areas of Katsina State, Nigeria. Infect Dis Poverty.

[53] Atalabi TE, Lawal U, Ipinlaye SJ (2016). Prevalence and intensity of genito-urinary schistosomiasis and associated risk factors among junior high school students in two local government areas around Zobe Dam in Katsina State, Nigeria. Parasit Vectors.

[54] Awosolu OB (2016). Epidemiology of urinary schistosomiasis and knowledge of health personnel in rural communities of South-Western Nigeria. J Parasitol Vector Biol.

[55] Awosolu OB, Akinnifesi OJ, Salawu AS, Omotayo YF, Obimakinde ET, Olise C (2019). Prevalence and intensity of urinary schistosomiasis among school age children in Ikota, Southwestern Nigeria. Braz J Biol Sci.

[56] Awosolu OB, Shariman YZ, Haziqah MTF, Olusi TA (2020). Will Nigerians win the war against urinary Schistosomiasis? Prevalence, intensity, risk factors and knowledge assessment among some rural communities in Southwestern Nigeria. Pathogens.

[57] Babagana U, Pukuma MS (2018). Epidemiology of schistosomiasis in Damboa, Gamboru and Baga (IDP) camps in Maiduguri, Borno state. Int J Res Publ..

[58] Balahbib A, Amarir F, Corstjens PL, de Dood CJ, van Dam GJ, Hajli A (2017). Selecting accurate post-elimination monitoring tools to prevent reemergence of urogenital schistosomiasis in Morocco: a pilot study. Infect Dis Poverty.

[59] Bangura ET, Ngegba MP, Nyalley F (2016). Prevalence and intensity of soil-transmitted helminthes (STHs) and schistosomes in primary schools in BO district, southern sierra Leone. Global J Biosci Biotechnol.

[60] Bashir SF, Usman U, Sani NM, Kawo AH (2016). Prevalence of *Schistosoma haematobium* among population Aged 1–25 years attending Rasheed Shekoni Specialist Hospital, Dutse, Jigawa State-Nigeria. J Pharm Biol Sci.

[61] Birma JS, Chessed G, Shadrach PA, Nganjiwa JI, Yako AB, Vandi P (2017). Urinary schistosomiasis in communities around Kiri Lake, Shelleng Local Government Area, Adamawa State, Nigeria. J Appl Sci Environ Manag.

[62] Bishop H, Akoh R (2018). Risk factors, symptoms and effects of urinary schistosomiasis on anthropometric indices of school children in Zaria, Kaduna state, Nigeria. Open Access J Sci.

[63] Bishop HG, Inabo HI, Ekah EE (2016). Prevalence and intensity of urinary schistosomiasis and their effects on packed cell volume of pupils in Jaba LGA, Nigeria. Edorium J Microbiol.

[64] Botelho MC, Machado A, Carvalho A, Vilaca M, Conceicao O, Rosa F (2016). *Schistosoma haematobium* in Guinea-Bissau: unacknowledged morbidity due to a particularly neglected parasite in a particularly neglected country. Parasitol Res.

[65] Boye A, Agbemator VK, Mate-Siakwa P, Essien-Baidoo S (2016). *Schistosoma haematobium* co-infection with soil-transmitted helminthes: prevalence and risk factors from two communities in the central region of Ghana. Int J Med Biomed Res.

[66] Dahesh S, Farid BE (2016). Epidemiological situation of urinary schistosomiasis in Tamwah area, Giza, Egypt: assessment and control. J Egypt Soc Parasitol.

[67] Dalhat M, Jibia AB, Mohammed D, Abdullahi S (2017). Intensity of urinary schistosomiasis on gender-aged group of primary schools children in Sokoto South and Kware Area, Sokoto State, Nigeria. Braz J Biol Sci.

[68] Damen JG, Kopkuk ED, Lugos MD (2018). Prevalence of urinary Schistosomiasis among irrigation farmers in North Central Nigeria. J Med Health Sci..

[69] Duwa RS, Sanusi A, Ogbunachara C, Okiemute F (2018). Prevalence of urinary Schistosomiasis among primary school children in three rural communities of Kano State, Nigeria. Niger Ann Pure Appl Sci.

[70] Ebong NE, Abah AE (2018). Preliminary studies on urinary Schistosomiasis in selected communities in Itu Local Government Area, Akwa Ibom State, Nigeria. J Pharm Biol Sci.

[71] Ekanem EE, Akapan FM, Eyong ME (2017). Urinary schistosomiasis in school children of a southern Nigerian community 8 years after the provision of potable water. Niger Postgrad Med J.

[72] Elhusein SI. Surveillance of Schistosoma species among population of Greater Wad Medani Locality, Gezira State, Sudan (2016). University of Gezira. 2016.

[73] Elom JE, Odikamnoro OO, Nnachi AU, Ikeh I, Nkwuda JO (2017). Variability of urine parameters in children infected with *Schistosoma haematobium* in Ukawu community, Onicha Local Government Area, Ebonyi State, Nigeria. Afr J Infect Dis.

[74] Elsiddig HA (2019). Prevalence of urinary schistosomiasis among schoolchildren in White Nile State, Sudan. Afr Educ Res J.

[75] Emmanuel OI, Agbo OE, Uche AJ, Odeh UP, Agogo IM (2017). Comparative evaluation of the prevalence of urinary schistosomiasis in two contrasting communities in Benue State, Nigeria. Int J Infect Dis Therapy.

[76] Fokuo Ofori M, Opoku Peprah B, Adukpo S, Kakra Dickson E, Anim-Baidoo I, Henry AR (2020). Prevalence of urinary and intestinal Schistosomiasis among rice framers in Asutsuare, Ghana. Int J Microbiol Biotechnol.

[77] Galappaththi-Arachchige HN, Amlie Hegertun IE, Holmen S, Qvigstad E, Kleppa E, Sebitloane M, et al. Association of urogenital symptoms with history of water contact in young women in areas endemic for *S. haematobium*. A cross-sectional study in Rural South Africa. Int J Environ Res Public Health. 2016;13(11). 10.3390/ijerph13111135 PMC5129345 27854250

[78] Galappaththi-Arachchige HN, Holmen S, Koukounari A, Kleppa E, Pillay P, Sebitloane M (2018). Evaluating diagnostic indicators of urogenital *Schistosoma haematobium* infection in young women: a cross sectional study in rural South Africa. PLoS ONE.

[79] Ganau AM, Mohammed K, Spencer THI, Nata’ala US, Kabiru Muhammad Asiya UI, Ibrahim G (2016). Intensity of urinary Schistosomiasis in relation to some epidemiologic markers in school children of Dundaye and Kwalkwalawa Riverine communities of Wamakko, Sokoto State, Nigeria. Sokoto J Med Lab Sci..

[80] Gbalegba NGC, Silue KD, Ba O, Ba H, Tian-Bi NTY, Yapi GY (2017). Prevalence and seasonal transmission of *Schistosoma haematobium* infection among school-aged children in Kaedi town, southern Mauritania. Parasit Vectors.

[81] Geraji JJ, Kator L, Hosea ZY. A survey of urinary Schistosomiasis among secondary school students in Jalingo Town, Jalingo Local Government Area, Taraba State. Asian J Res Zool. 2019:1–6.

[82] Ghieth MA, Lotfy AM (2017). Schistosomiasis haematobium prevalence among haematuric patients: parasitological and immuno-assay. Beni-Suef Univ J Basic Appl Sci.

[83] Goodhead DA, Dirisu CG (2016). Prevalence of urinary schistosomiasis among pupils in endemic communities of Rivers state, Nigeria. Am J Microbiol Biotechnol.

[84] Greter H, Krauth SJ, Ngandolo BN, Alfaroukh IO, Zinsstag J, Utzinger J (2016). Validation of a point-of-care circulating cathodic antigen urine cassette test for *Schistosoma mansoni* Diagnosis in the Sahel, and Potential Cross-Reaction in Pregnancy. Am J Trop Med Hyg.

[85] Hajissa K, Muhajir A, Eshag HA, Alfadel A, Nahied E, Dahab R (2018). Prevalence of schistosomiasis and associated risk factors among school children in Um-Asher Area, Khartoum, Sudan. BMC Res Notes.

[86] Houmsou RS, Agere H, Wama BE, Bingbeng JB, Amuta EU, Kela SL (2016). Urinary Schistosomiasis among children in Murbai and Surbai communities of Ardo-Kola Local Government Area, Taraba State. Nigeria J Trop Med.

[87] IDRIS M, ISAH AU. Incidence of urinary schistosomiasis among rice farmers in selected villages in Borgu Local Government Area of Niger State, Nigeria. NISEB J. 2019;11(2).

[88] Igbeneghu C, Onuegbu JA, Olisekodiaka JM, Alabi T (2016). Urinary schistosomiasis among school pupils in Ilie community, Southwestern Nigeria. Saudi J Med Pharm Sci.

[89] Kabuyaya M, Chimbari MJ, Manyangadze T, Mukaratirwa S (2017). Schistosomiasis risk factors based on the infection status among school-going children in the Ndumo area, uMkhanyakude district, South Africa. South Afr J Infect Dis.

[90] Kaiglová A, Changoma MJS, Špajdelová J, Jakubcová D, Bírová K (2020). Urinary schistosomiasis in patients of rural medical health centers in Kwale county, Kenya. Helminthologia.

[91] Kayuni S, Peeling R, Makaula P (2017). Prevalence and distribution of *Schistosoma haematobium* infection among school children living in southwestern shores of Lake Malawi. Malawi Med J.

[92] Kenneth IE, Itohan IM, Godwin NO (2017). Bacteriuria and urinary Schistosomiasis among individuals in Ewean community Akoko—Edo, Edo State, Nigeria. Am J Microbiol Biotechnol.

[93] Maki A, Ali GA, Hajissa K. Prevalence and intensity of urinary Schistosomiasis among selected people in Tulus Area, South Darfur State, Sudan. 2020:2–8.

[94] Mohammed K, Hassan J, Opaluwa SA, Adamu T, Spencer THI, Aschroft OF (2018). Prevalence of urinary Schistosomiasis among school-age children in Kashinzama and Sabiyal in Aliero Local Government Area, Kebbi State, Nigeria. South Asian J Parasitol.

[95] Mohammed K, Suwaiba M, Spencer T, Nataala S, Ashcroft O, Nuhu A (2018). Prevalence of urinary Schistosomiasis among primary school children in Kwalkwalawa Area, Sokoto State, North-Western Nigeria. Asian J Res Med Pharm Sci.

[96] Mohammed MK, Halaly S, Awadalla H, Abdelrahman A, Balla S (2018). Prevalence, risk factors and effect of urinary Schistosomiasis on academic performance of school children age 6–15 years in Asalaya Locality, White Nile State, Sudan 2017. J Adv Med Med Res.

[97] Mombo-Ngoma G, Honkpehedji J, Basra A, Mackanga JR, Zoleko RM, Zinsou J (2017). Urogenital schistosomiasis during pregnancy is associated with low birth weight delivery: analysis of a prospective cohort of pregnant women and their offspring in Gabon. Int J Parasitol.

[98] Mong K, Chikodi S, Ihemanma CA (2016). Current prevalence status of urinary Schistosomiasis among children in Lokpanta Community, Abia State, Nigeria. Galore Int J Health Sci Res.

[99] Morenikeji OA, Eleng IE, Atanda OS, Oyeyemi OT (2016). Renal related disorders in concomitant *Schistosoma haematobium*-*Plasmodium falciparum* infection among children in a rural community of Nigeria. J Infect Public Health.

[100] Moyo VB, Changadeya W, Chiotha S, Sikawa D (2016). Urinary schistosomiasis among preschool children in Malengachanzi, Nkhotakota District, Malawi: prevalence and risk factors. Malawi Med J.

[101] Muhammad IA, Abdullahi K, Bala AY, Shinkafi SaA. Prevalence of urinary schistosomiasis among primary school pupils in Wamakko Local Government, Sokoto State, Nigeria. J Basic Appl Zool. 2019;80(1).

[102] Musa NY, Dadah AJ, Auwalu U (2016). Prevalence of urinary schistosomiasis among secondary school students in Chikun local government area, Kaduna state, Nigeria. Int J Sci Eng Res.

[103] Mutsaka-Makuvaza MJ, Matsena-Zingoni Z, Katsidzira A, Tshuma C, Chin'ombe N, Zhou XN (2019). Urogenital schistosomiasis and risk factors of infection in mothers and preschool children in an endemic district in Zimbabwe. Parasit Vectors.

[104] Nafiu S, Inuwa B, Abdullahi A, Alkali Z, Ibrahim BA (2016). Prevalence of urinary schistosomiasis among primary school pupils in Kofa primary school, Tafa local government, Niger state, Nigeria. Ewemen J Epidemiol Clin Med.

[105] Ngwamah JS, Naphtali RS. Prevalence and intensity of urinary Schistosomiasis among residence: a case study in River Benue, Adamawa State, North Eastern Nigeria. Asian J Res Zool. 2019:1–10.

[106] Noriode RM, Idowu ET, Otubanjo OA, Mafe MA (2018). Urinary schistosomiasis in school aged children of two rural endemic communities in Edo State, Nigeria. J Infect Public Health.

[107] Nwachukwu IO, Ukaga CN, Ajero CMU, Nwoke BEB, Nwachukwu MI, Obasi CC (2018). Urinary Schistosomiasis and concomitant Bacteriuria among school age children in some parts of Owerri, Imo State. Int Res J Adv Eng Sci.

[108] Nwachukwu PC, Ohaeri CC, Ukpai OM, Irole-eze OP, Amaechi EC (2018). Prevalence of *Schistosoma haematobium* infection among school-aged children in Afikpo North local government area, Ebonyi State, Nigeria. Sri Lankan J Biol.

[109] Nyarko R, Torpey K, Ankomah A (2018). *Schistosoma haematobium*, *Plasmodium falciparum* infection and anaemia in children in Accra. Ghana Trop Dis Travel Med Vaccines.

[110] Obisike VU, Amuta EU, Audu AB, Kwenev SA (2019). Comparison of polycarbonate filter paper and sedimentation methods in diagnosing *Schistosoma haematobium* infection in Makurdi, Benue, Nigeria. South Asian J Parasitol.

[111] Oladeinde B, Okpala O, Onifade A, Osaiyuwu O, Ayoola A (2018). Urinary schistosomiasis: a study among primary school pupils in a rural community in Nigeria. Trop J Health Sci.

[112] Olayinka P, Ajide P, Awobode HO, Osundiran AJ, Onile OS, Adebayo AS (2020). Co-infection of schistosomiasis, malaria, HBV and HIV among adults living in Eggua Community, Ogun State, Nigeria. Nigerian J Parasitol.

[113] Oluwatoyin AH, Olukemi OD, Omolara OA, Adetola AT (2016). Prevalence of Schistosoma and other parasites among female residents of some communities in Oyo state, Nigeria. J Public Health Epidemiol.

[114] Oluwole AS, Adeniran AA, Mogaji HO, Olabinke DB, Abe EM, Bankole SO, et al. Prevalence, intensity and spatial co-distribution of schistosomiasis and soil transmitted helminths infections in Ogun state, Nigeria. Parasitol Open. 2018;4.

[115] Onile OS, Awobode HO, Oladele VS, Agunloye AM, Anumudu CI (2016). Detection of urinary tract pathology in some *Schistosoma haematobium* infected Nigerian adults. J Trop Med.

[116] Orpin JB, Bem AA, Usman A (2017). Prevalence of urinary schistosomiasis in selected secondary school students of Faskari Local Government Area, Katsina State. FUDMA J Sci (FJS).

[117] Orpin JB, Manyi MM, Bem AA, Mzungu I (2016). Prevalence of Urinary schistosomiasis in Oju Local Government Area of Benue State Nigeria. FUDMA J Sci Educ Res.

[118] Otuneme OG, Obebe OO, Sajobi TT, Akinleye WA, Faloye TG (2019). Prevalence of schistosomiasis in a neglected community, South western Nigeria at two points in time, spaced three years apart. Afr Health Sci.

[119] Paul CI, Aniedi ED, Ofonime MO, Uloma O (2017). Urogenital schistosomiasis and intestinal parasitosis coinfection among school age children in Adim community Nigeria. Int J Sci.

[120] Phillips AE, Gazzinelli-Guimaraes PH, Aurelio HO, Dhanani N, Ferro J, Nala R (2018). Urogenital schistosomiasis in Cabo Delgado, northern Mozambique: baseline findings from the SCORE study. Parasit Vectors.

[121] Pillay P, Taylor M, Van Lieshout L, Roald B (2016). Female genital schistosomiasis (fgs) as a risk factor for squamous cell atypia in an epidemiological longitudinal cohort of young women in Kwazulu-natal.

[122] Qutoof HashimTaha OHE, Ahmed AI (2019). Distribution of urinary schistosomiasis among school children at elkeriab and tayba elkababish villages, East Nile Locality, Khartoum State, Sudan. J Pediatr Neonatal Care..

[123] Rasoamanamihaja CF, Rahetilahy AM, Ranjatoarivony B, Dhanani N, Andriamaro L, Andrianarisoa SH (2016). Baseline prevalence and intensity of schistosomiasis at sentinel sites in Madagascar: informing a national control strategy. Parasit Vectors.

[124] Sabah Alzain Mohamed H, Elamin A (2017). Detection rate of urinary schistosomiasis in El khiar Villages White Nile State, Sudan. Pyrex J Biomed Res..

[125] Senghor B, Diaw OT, Doucoure S, Seye M, Diallo A, Talla I (2016). Impact of annual praziquantel treatment on urogenital schistosomiasis in a seasonal transmission focus in Central Senegal. PLoS Negl Trop Dis.

[126] Shukla JD, Kleppa E, Holmen S, Ndhlovu PD, Mtshali A, Sebitloane MH, et al. Female genital schistosomiasis and reproductive tract infections. A cross-sectional study in rural adolescents in South Africa. medRxiv. 2019:19009233. 10.1097/LGT.0000000000000756 PMC10309100 37379442

[127] Simoonga C, Kazembe LN (2017). Using the hierarchical ordinal regression model to analyse the intensity of urinary schistosomiasis infection in school children in Lusaka Province, Zambia. Infect Dis Poverty.

[128] Sobande AI, Morenikeji O, Emikpe BO, Akinboade OA, Adewoga TOS. Prevalence and Intensity of urinary schistosomiasis in school-age children in Yewa North Local Government Area of Ogun State, Nigeria. Ann Res Rev Biol. 2019:1–6.

[129] Sule H, Kumurya AS, Mansur MH (2019). urinary schistosomiasis among primary school pupils in Dawakin kudu local government area, Kano state Fudma. J Sci.

[130] Sulieman Y, Eltayeb RE, Pengsakul T, Afifi A, Zakaria MA (2017). Epidemiology of Urinary Schistosomiasis among School Children in the Alsaial Alsagair Village, River Nile State, Sudan. Iran J Parasitol.

[131] Talab H, Kardaman M, Alhidai S, Eissa M. Assessment of diagnostic methods for urinary schistosomiasis, Assalya, White Nile State, Sudan. Eur Acad Res. 2018;5(10).

[132] Umar M, Umar U, Usman I, Yahaya A, Dambazau S (2016). *Schistosoma haematobium* infections: prevalence and morbidity indicators in communities around Wasai Dam, Minjibir, Kano State, Northern Nigeria. Int J Trop Dis Health.

[133] Usman AS, Malann YD, Babeker EA (2016). Prevalence of *Schistosoma haematobium* among School Children in Bauchi State, Nigeria. Int J Innov Sci Res.

[134] Wilkinson JP (2018). Schistosomiasis among obstetric fistula patients in Lilongwe, Malawi. Malawi Med J.

[135] Wokem GN, Edache Abah A, Ukuku EO (2017). Infection status of school children with *Schistosoma haematobium* in an urban setting in South-eastern Nigeria. Zool Ecol.

[136] Woldegerima E, Bayih AG, Tegegne Y, Aemero M, Jejaw ZA (2019). Prevalence and reinfection rates of *Schistosoma mansoni* and praziquantel efficacy against the parasite among primary school children in Sanja Town, Northwest Ethiopia. J Parasitol Res.

[137] Yameny AA (2018). Schistosomiasis haematobium prevalence and risk factors in EL-Fayoum Governorate, Egypt. J Biosci Appl Res.

[138] Yauba SMRA, Farouk AG, Elechi HA, Ummate I, Ibrahim BA, Ibrahim HA, Baba AS, Boda TA, Olowu WA (2018). Urinary schistosomiasis in Boko Haram-related internally displaced Nigerian children. Saudi J Kidney Dis Transplant.

[139] Zida A, Briegel J, Kabré I, Sawadogo MP, Sangaré I, Bamba S (2016). Epidemiological and clinical aspects of urogenital schistosomiasis in women, in Burkina Faso, West Africa. Infect Dis Poverty.

[140] Stutzer C, Richards SA, Ferreira M, Baron S, Maritz-Olivier C (2018). Metazoan parasite vaccines: present status and future prospects. Front Cell Infect Microbiol.

[141] Di Bella S, Riccardi N, Giacobbe DR, Luzzati R (2018). History of schistosomiasis (bilharziasis) in humans: from Egyptian medical papyri to molecular biology on mummies. Pathogens Global Health.

[142] Steinmann P, Keiser J, Bos R, Tanner M, Utzinger J (2006). Schistosomiasis and water resources development: systematic review, meta-analysis, and estimates of people at risk. Lancet Infect Dis.

[143] Ezeh CO, Onyekwelu KC, Akinwale OP, Shan L, Wei H. Urinary schistosomiasis in Nigeria: a 50 year review of prevalence, distribution and disease burden. Parasite. 2019;26. 10.1051/parasite/2019020 PMC6447092 30943149

[144] Nelson G, Teesdale C, Highton R, editors. The role of animals as reservoirs of bilharziasis in Africa. Ciba Foundation Symposium‐Bilharziasis; 1962: Wiley Online Library.

[145] Adamson P (1976). Schistosomiasis in antiquity. Med Hist.

[146] Burke M, Jones M, Gobert G, Li Y, Ellis M, McManus D (2009). Immunopathogenesis of human schistosomiasis. Parasite Immunol.

[147] Fairfax K, Nascimento M, Huang SC-C, Everts B, Pearce EJ, editors. Th2 responses in schistosomiasis. Seminars in immunopathology; 2012: Springer. 10.1007/s00281-012-0354-4 23139101

[148] Pearce EJ, MacDonald AS (2002). The immunobiology of schistosomiasis. Nat Rev Immunol.

[149] Peterson WP, von Lichtenberg F (1965). Studies on granuloma formation. IV: in vivo antigenicity of schistosome egg antigen in lung tissue. J Immunol.

[150] King CH, Keating CE, Muruka JF, Ouma JH, Houser H, Siongok TKA (1988). Urinary tract morbidity in schistosomiasis haematobia: associations with age and intensity of infection in an endemic area of Coast Province, Kenya. Am J Trop Med Hyg.

[151] Wamachi AN, Mayadev JS, Mungai PL, Magak PL, Ouma JH, Magambo JK (2004). Increased ratio of tumor necrosis factor-α to interleukin-10 production is associated with *Schistosoma haematobium*-induced urinary-tract morbidity. J Infect Dis.

[152] Khalaf I, Shokeir A, Shalaby M (2012). Urologic complications of genitourinary schistosomiasis. World J Urol.

[153] Schwartz D (1981). Helminths in the induction of cancer II. *Schistosoma haematobium* and bladder cancer. Trop Geogr Med.

[154] Figueiredo JC, Richter J, Borja N, Balaca A, Costa S, Belo S (2015). Prostate adenocarcinoma associated with prostatic infection due to *Schistosoma haematobium*. Case report and systematic review. Parasitol Res.

[155] Richardson ML, Fu C-L, Pennington LF, Honeycutt JD, Odegaard JL, Hsieh Y-J (2014). A new mouse model for female genital schistosomiasis. PLoS Negl Trop Dis.

[156] Talaat M, Watts S, Mekheimar S, Ali HF, Hamed H (2004). The social context of reproductive health in an Egyptian hamlet: a pilot study to identify female genital schistosomiasis. Soc Sci Med.

[157] Sturt A, Webb E, Francis S, Hayes R, Bustinduy A. Beyond the barrier: female genital schistosomiasis as a potential risk factor for HIV-1 acquisition. Acta Trop. 2020:105524. 10.1016/j.actatropica.2020.105524 PMC7429987 32416076

[158] Patel P, Rose CE, Kjetland EF, Downs JA, Mbabazi PS, Sabin K, et al. Association of schistosomiasis and HIV infections: a systematic review and meta-analysis. Int J Infect Dis. 2020. 10.1016/j.ijid.2020.10.088 PMC8883428 33157296

[159] Ribeiro AR, Luis C, Fernandes R, Botelho MC (2019). Schistosomiasis and infertility: what do we know?. Trends Parasitol.

[160] Chadeka EA, Nagi S, Sunahara T, Cheruiyot NB, Bahati F, Ozeki Y (2017). Spatial distribution and risk factors of *Schistosoma haematobium* and hookworm infections among schoolchildren in Kwale, Kenya. PLoS Negl Trop Dis.

[161] Corstjens PL, Hoekstra PT, Claudia J, van Dam GJ (2017). Utilizing the ultrasensitive Schistosoma up-converting phosphor lateral flow circulating anodic antigen (UCP-LF CAA) assay for sample pooling-strategies. Infect Dis Poverty.

[162] Gray DJ, Ross AG, Li Y-S, McManus DP (2011). Diagnosis and management of schistosomiasis. BMJ.

[163] Weerakoon KG, Gobert GN, Cai P, McManus DP (2015). Advances in the diagnosis of human schistosomiasis. Clin Microbiol Rev.

[164] Bishop H, Akoh R (2016). Risk factors, symptoms and effects of urinary schistosomiasis on anthropometric indices of school children in Zaria, Kaduna state, Nigeria. Open Access J Sci.

[165] Nwibari BMW, Johnson CT, Yohanna JA, Dakul DA (2016). Prevalence of urinary schistosomiasis among human immunodeficiency virus patients attending faith alive medical centre in Jos North, Plateau State, Nigeria. Int J Biomed Health Sci.

[166] Omoruyi Z, Enoruwa UD (2016). Urinary schistosomiasis among primary and junior secondary school children in Uhunmwode Local Government Area of Edo State. J Med Biomed Res.

[167] Talab H, Kardaman M, Alhidai S, Eissa M, Bayoumi M. Assessment of diagnostic methods for urinary schistosomiasis, Assalya, White Nile State, Sudan. Eur Acad Res. 2018;5(10).

[168] Galappaththi-Arachchige HN, Amlie Hegertun IE, Holmen S, Qvigstad E, Kleppa E, Sebitloane M (2016). Association of urogenital symptoms with history of water contact in young women in areas endemic for *S. haematobium*. A cross-sectional study in Rural South Africa. Int J Environ Res Public Health.

[169] Pillay P, van Lieshout L, Taylor M, Sebitloane M, Zulu SG, Kleppa E (2016). Cervical cytology as a diagnostic tool for female genital schistosomiasis: correlation to cervical atypia and Schistosoma polymerase chain reaction. CytoJournal..

