# Supplementary material for: Prevalence of urinary schistosomiasis in women: a systematic review and meta-analysis of recently published literature (2016–2020)
Source: Trop Med Health. 2022 Jan 29;50:12. doi: 10.1186/s41182-022-00402-x (PMC8800356; doi:10.1186/s41182-022-00402-x)
Supplement: Supplementary file 2 — Additional file 2. Forest plot of the FUS prevalence obtained from published literature during 2016–2020. [file 41182_2022_402_MOESM2_ESM.docx]

**Supplementary Fig. 1.** Forest plot of the FUS prevalence obtained from published literature during 2016-2020.
