# Supplementary material for: Prevalence of urinary schistosomiasis in women: a systematic review and meta-analysis of recently published literature (2016–2020)
Source: Trop Med Health. 2022 Jan 29;50:12. doi: 10.1186/s41182-022-00402-x (PMC8800356; doi:10.1186/s41182-022-00402-x)
Supplement: Supplementary file 3 — Additional file 3. Quality assessment analysis of the included papers using Newcastle–Ottawa scale. [file 41182_2022_402_MOESM3_ESM.docx]

**Additional File 3.** Qualitative analysis of each included study using Newcastle-Ottawa scale for cross-sectional studies.

|  | **Selection** | | | | | **Comparability** | **Outcome** | |  |
| --- | --- | --- | --- | --- | --- | --- | --- | --- | --- |
| **Study** | **Representativeness of the sample** | **Sample size** | **Non-respondents** | | **Ascertainment of the exposure (risk factor)** | **Confounding factors controlled** | **Assessment of outcome** | **Statistical test** | **Total quality score** |
| Awosolu 2020 | 1 | 1 | | 1 | 0 | 0 | 1 | 1 | 5 |
| Olayinka 2020 | 1 | 1 | | 1 | 1 | 0 | 1 | 1 | 6 |
| Awosolu 2019 | 1 | 1 | | 1 | 1 | 1 | 1 | 1 | 7 |
| Otuneme 2019 | 1 | 1 | | 1 | 0 | 1 | 0 | 1 | 5 |
| Muhammad 2019 | 1 | 1 | | 1 | 0 | 1 | 0 | 1 | 5 |
| Sule 2019 | 1 | 1 | | 1 | 1 | 0 | 1 | 1 | 6 |
| Idris and Isah 2019 | 1 | 1 | | 1 | 1 | 1 | 1 | 1 | 7 |
| Geraji 2019 | 1 | 1 | | 1 | 1 | 1 | 1 | 1 | 7 |
| Adamu 2019 | 1 | 1 | | 1 | 1 | 1 | 1 | 1 | 7 |
| Ngwamah and Naphtali 2019 | 1 | 1 | | 1 | 1 | 1 | 1 | 1 | 7 |
| Aribodor 2019 | 1 | 1 | | 1 | 1 | 1 | 1 | 1 | 7 |
| Sobande 2019 | 1 | 1 | | 1 | 1 | 1 | 0 | 1 | 6 |
| Obisike 2019 | 1 | 1 | | 1 | 1 | 0 | 0 | 1 | 5 |
| Ahmed and Gidado 2019 | 1 | 1 | | 1 | 1 | 0 | 1 | 1 | 6 |
| Aderibigbe 2019 | 1 | 1 | | 1 | 1 | 1 | 1 | 1 | 7 |
| Noriode 2018 | 1 | 1 | | 1 | 0 | 0 | 1 | 1 | 5 |
| Bishop and Akoh 2016 | 1 | 1 | | 1 | 1 | 0 | 1 | 1 | 6 |
| Mohammed 2018b | 1 | 1 | | 1 | 0 | 0 | 1 | 1 | 5 |
| Akinneye 2018 | 1 | 1 | | 1 | 1 | 0 | 0 | 1 | 5 |
| Alabi 2018 | 1 | 1 | | 1 | 1 | 1 | 0 | 1 | 6 |
| Damen 2018 | 1 | 1 | | 1 | 1 | 0 | 1 | 1 | 6 |
| Yauba 2018 | 1 | 1 | | 1 | 1 | 1 | 1 | 1 | 7 |
| Abdulkareem 2018 | 1 | 1 | | 1 | 1 | 1 | 1 | 1 | 7 |
| Oladeinde 2018 | 1 | 1 | | 1 | 1 | 0 | 1 | 1 | 6 |
| Ebong and Abah 2018 | 1 | 1 | | 1 | 1 | 1 | 1 | 1 | 7 |
| Adebayo and Oladimeji 2018 | 1 | 1 | | 1 | 0 | 0 | 1 | 1 | 5 |
| Oluwole 2018 | 1 | 1 | | 1 | 0 | 1 | 1 | 1 | 6 |
| Adewale 2018 | 1 | 1 | | 1 | 0 | 1 | 1 | 1 | 6 |
| Nwachukwu 2018a | 1 | 1 | | 1 | 1 | 1 | 1 | 1 | 7 |
| Nwachukwu 2018b | 1 | 1 | | 1 | 1 | 1 | 1 | 1 | 7 |
| Duwa 2018 | 1 | 1 | | 1 | 0 | 1 | 0 | 1 | 5 |
| Babagana and pukuma 2018 | 1 | 1 | | 1 | 1 | 1 | 1 | 1 | 7 |
| Mohammed 2018a | 1 | 1 | | 1 | 1 | 0 | 0 | 1 | 5 |
| Oluwole 2018 | 1 | 1 | | 1 | 1 | 0 | 1 | 1 | 6 |
| Kenneth 2017 | 1 | 1 | | 1 | 1 | 1 | 1 | 1 | 7 |
| Birma 2017 | 1 | 1 | | 1 | 0 | 1 | 0 | 1 | 5 |
| Amoo 2017 | 1 | 1 | | 1 | 0 | 1 | 1 | 1 | 6 |
| Paul 2017 | 1 | 1 | | 1 | 0 | 0 | 1 | 1 | 5 |
| Orpin 2017 | 1 | 1 | | 1 | 0 | 0 | 1 | 1 | 5 |
| Ekanem 2017 | 1 | 1 | | 1 | 0 | 1 | 1 | 1 | 6 |
| Akpan and Ejezie 2017 | 1 | 1 | | 1 | 1 | 1 | 1 | 1 | 7 |
| Elom 2017 | 1 | 1 | | 1 | 1 | 1 | 1 | 1 | 7 |
| Akpan 2017 | 1 | 1 | | 1 | 1 | 1 | 1 | 1 | 7 |
| Abubakar 2017 | 1 | 1 | | 1 | 1 | 1 | 1 | 1 | 7 |
| Dalhat 2017 | 1 | 1 | | 1 | 1 | 1 | 1 | 1 | 7 |
| Emmanuel 2017 | 1 | 1 | | 1 | 1 | 0 | 1 | 1 | 6 |
| Wokem 2017 | 1 | 1 | | 1 | 1 | 1 | 1 | 1 | 7 |
| Anorue 2017 | 1 | 1 | | 1 | 1 | 0 | 1 | 1 | 6 |
| Orpin 2016 | 1 | 1 | | 1 | 1 | 1 | 1 | 1 | 7 |
| Onile 2016 | 1 | 1 | | 1 | 1 | 1 | 1 | 1 | 7 |
| Houmsou 2016 | 1 | 1 | | 1 | 0 | 0 | 1 | 1 | 5 |
| Awosolu 2016 | 1 | 1 | | 1 | 0 | 0 | 1 | 1 | 5 |
| Goodhead and Dirisu 2016 | 1 | 1 | | 1 | 1 | 1 | 1 | 1 | 7 |
| Usman 2016 | 1 | 1 | | 1 | 1 | 1 | 1 | 1 | 7 |
| Dahesh and Farid 2016 | 1 | 1 | | 1 | 1 | 1 | 1 | 1 | 7 |
| Igbeneghu 2016 | 1 | 1 | | 1 | 1 | 1 | 1 | 1 | 7 |
| Nafiu 2016 | 1 | 1 | | 1 | 1 | 0 | 1 | 1 | 6 |
| Abah 2016 | 1 | 1 | | 1 | 0 | 0 | 1 | 1 | 5 |
| Umar 2016 | 1 | 1 | | 1 | 0 | 1 | 0 | 1 | 5 |
| Atalabi 2016a | 1 | 1 | | 1 | 1 | 0 | 1 | 1 | 6 |
| Houmsou 2016 | 1 | 1 | | 1 | 1 | 1 | 1 | 1 | 7 |
| Nwibari 2016 | 1 | 1 | | 1 | 1 | 0 | 1 | 1 | 5 |
| Omoroyi and Enoruwa 2016 | 1 | 1 | | 1 | 1 | 0 | 1 | 1 | 6 |
| Morenikeji 2016 | 1 | 1 | | 1 | 1 | 0 | 1 | 1 | 6 |
| Bashir 2016 | 1 | 1 | | 1 | 1 | 1 | 1 | 1 | 7 |
| Ganau 2016 | 1 | 1 | | 1 | 0 | 0 | 1 | 1 | 5 |
| Musa 2016 | 1 | 1 | | 1 | 0 | 1 | 1 | 1 | 6 |
| Ajakaye 2016 | 1 | 1 | | 1 | 1 | 1 | 1 | 1 | 7 |
| Mong 2016 | 1 | 1 | | 1 | 1 | 1 | 1 | 1 | 7 |
| Atalabi 2016b | 1 | 1 | | 1 | 1 | 0 | 1 | 1 | 6 |
| Oluwatoyin 2016 | 1 | 1 | | 1 | 1 | 1 | 1 |  | 7 |
| Oluwatoyin 2016 | 1 | 1 | | 1 | 0 | 1 | 1 | 1 | 6 |
| Bishop 2016 | 1 | 1 | | 1 | 0 | 1 | 0 | 1 | 5 |
| Maki 2020 | 1 | 1 | | 1 | 1 | 0 | 1 | 1 | 6 |
| Qutoof 2019 | 1 | 1 | | 1 | 1 | 0 | 0 | 1 | 5 |
| Elsiddig 2019 | 1 | 1 | | 1 | 0 | 1 | 1 | 1 | 6 |
| Hajissa 2018 | 1 | 1 | | 1 | 0 | 1 | 1 | 1 | 6 |
| Mohammed 2018c | 1 | 1 | | 1 | 1 | 1 | 1 | 1 | 7 |
| Talab 2018 | 1 | 1 | | 1 | 0 | 0 | 1 | 1 | 5 |
| Suleiman 2017 | 1 | 1 | | 1 |  | 0 | 1 | 1 | 6 |
| Sabah Alzain and Elamin 2017 | 1 | 1 | | 1 | 0 | 0 | 1 | 1 | 5 |
| Afifi 2016 | 1 | 1 | | 1 | 0 | 1 | 1 | 1 | 6 |
| Elhusein 2016 | 1 | 1 | | 1 | 1 | 1 | 1 | 1 | 7 |
| Shukla 2019 | 1 | 1 | | 1 | 0 | 1 | 0 | 1 | 5 |
| Galappaththi-Arachchige 2018 | 1 | 1 | | 1 | 0 | 1 | 0 | 1 | 5 |
| Kabuyaya 2017 | 1 | 1 | | 1 | 1 | 1 | 1 | 1 | 7 |
| Galappaththi-Arachchige 2016 | 1 | 1 | | 1 | 1 | 0 | 1 | 1 | 6 |
| Pillay 2016 | 1 | 1 | | 1 | 1 | 1 | 1 | 1 | 7 |
| Pillay 2016 | 1 | 1 | | 1 | 1 | 1 | 1 | 1 | 7 |
| Pillay 2016 | 1 | 1 | | 1 | 1 | 1 | 1 | 1 | 7 |
| Foku ofori 2020 | 1 | 1 | |  | 1 | 0 | 1 | 1 | 6 |
| Arhin-Wiredu 2019 | 1 | 1 | | 1 | 1 | 0 | 1 | 1 | 6 |
| Nyarko 2018 | 1 | 1 | |  | 1 | 0 | 1 | 1 | 6 |
| Boye 2016 | 1 | 1 | | 1 | 0 | 0 | 1 | 1 | 5 |
| Wilkinson 2018 | 1 | 1 | | 1 | 0 | 1 | 1 | 1 | 6 |
| Kayuni 2017 | 1 | 1 | | 1 | 0 | 1 | 1 | 1 | 6 |
| Moyo 2016 | 1 | 1 | | 1 | 0 | 1 | 1 | 1 | 6 |
| Yameny 2018 | 1 | 1 | | 1 | 1 | 1 | 1 | 1 | 7 |
| Ghieth and Lotfy 2017 |  | 1 | | 1 | 0 | 0 | 1 | 1 | 5 |
| Kaiglova 2020 | 1 | 1 | | 1 | 0 | 0 | 1 | 1 | 5 |
| Makuvaza 2019 | 1 | 1 | | 1 | 1 | 0 | 1 | 1 | 6 |
| Woldegerima 2019 | 1 | 1 | | 1 | 1 | 1 | 1 | 1 | 7 |
| Philips 2018 | 1 | 1 | | 1 | 1 | 1 | 1 | 1 | 7 |
| Gbalegba 2017 | 1 | 1 | | 1 | 1 | 0 | 1 | 1 | 6 |
| Simoonga and Kazembe 2017 | 1 | 1 | | 1 | 1 | 1 | 1 | 1 | 7 |
| Balahbib 2017 | 1 | 1 | | 1 | 1 | 0 | 1 | 1 | 6 |
| Anchang-Kimbi 2017 | 1 | 1 | | 1 | 1 | 1 | 1 | 1 | 7 |
| Mombo-Ngoma 2017 | 1 | 1 | | 1 | 1 | 1 | 1 | 1 | 7 |
| Greter 2016 | 1 | 1 | | 1 | 1 | 1 | 1 | 1 | 7 |
| Botelho 2016 | 1 | 1 | | 1 | 1 | 0 | 1 | 1 | 6 |
| Senghor 2016 | 1 |  | | 1 | 0 | 0 | 1 | 1 | 5 |
| Rasomanamihaja 2016 | 1 | 1 | | 1 | 0 | 0 | 1 | 1 | 5 |
| Bangura 2016 | 1 | 1 | | 1 | 1 | 1 | 1 | 1 | 7 |
| Zida 2016 | 1 | 1 | | 1 | 1 | 1 | 1 | 1 | 7 |
